# Unearthing prehistoric diets: First evidence of horse meat consumption in Early Bronze Age Sicily

**DOI:** 10.1371/journal.pone.0330772

**Published:** 2025-08-29

**Authors:** Davide Tanasi, Roberto Micciché, Robert H. Tykot, Luis Busetti, Pierluigi Barbieri, Gianpiero Di Maida, Viviana Ardesia, Alessandro Miani, Elia Marin, Enrico Greco

**Affiliations:** 1 Department of History, University of South Florida, Tampa, Florida, United States of America; 2 Department of Culture and Societies, University of Palermo, Palermo, Italy; 3 Department of Anthropology, University of South Florida, Tampa, Florida, United States of America; 4 Department of Chemical and Pharmaceutical Sciences, University of Trieste, Trieste, Italy; 5 Lower Saxony State Office for Cultural Heritage, Hannover, Germany; 6 Department of Languages and Linguistics, University of Tampa, Tampa, Florida, United States of America; 7 Società Italiana di Medicina Ambientale (SIMA), Milano, Italy; 8 Biomedical Engineering Laboratory, Kyoto Institute of Technology, Matsugasaki, Kyoto, Japan; 9 Research Institute of the University of Bucharest (ICUB), University of Bucharest, Bucharest, Romania; 10 Institute for the Advanced Study of Culture and the Environment (IASCE), University of South Florida, Tampa, Florida, United States of America; Tel Aviv university, ISRAEL

## Abstract

This paper presents the earliest documented evidence for the presence and consumption of horse meat in Early Bronze Age Sicily, significantly revising previous understandings of equid use on the island. Multidisciplinary analyses involving proteomics and lipidomics were performed on ceramic vessels from the Castelluccian settlement at Polizzello Mountain (Caltanissetta), revealing residues consistent with equine-derived substances. Proteomic data unequivocally identified equine serum albumin in multiple pottery fragments, demonstrating active consumption or processing of horse-derived substances within a ceremonial or dietary context. Lipid residues further supported this interpretation, indicating the presence of animal fats and vegetable-derived substances within the pottery. These findings substantially alter existing models of horse domestication, utilization, and dietary practices in prehistoric Sicily, suggesting a far earlier and more complex human-equid relationship. Furthermore, the integration of biomolecular data enhances our understanding of intercultural interactions, ritual behaviors, and economic strategies in the central Mediterranean during the third millennium BCE.

## 1. Introduction

Within the framework of human societal evolution, equids, especially the horse (*Equus caballus* Linnaeus, 1758) and the donkey (*Equus asinus* Linnaeus, 1758), played a vital role in shaping and developing the economic and cultural structures of the ancient world. From the earliest documented interactions between humans and equids, these animals constituted a strategic resource, profoundly influencing subsistence patterns, settlement models, transportation systems, agricultural practices, and military strategies. From an economic perspective, horses and donkeys performed complementary functions. The donkey, resilient and economical in terms of resource consumption, became indispensable for the transport of heavy loads in arid and mountainous regions, thus facilitating the establishment of the earliest trade routes. By contrast, the horse, with its speed and endurance, revolutionized human mobility and became an essential element within aristocratic and military elites [1–3].

Zooarchaeology, through the analysis of osteological remains, iconographic representations, and written sources, has demonstrated that human-equid relationships evolved progressively and in complex ways. These interactions shifted from the hunting of wild equid forms during the Lower and Middle Paleolithic periods [4,5] to their systematic domestication during the Eneolithic and the Bronze Age. This process is evidenced by significant finds at sites on the Eurasian steppe associated with the Botai and Yamnaya cultures, where the earliest evidence of horse domestication dates to the 4^th^–3^rd^ millennium BCE [2,6], as well as by discoveries at Maadi [7] and Abydos [8] in Egypt, documenting the use of the donkey as a pack animal already by the 4^th^ millennium BCE.

Despite the well-documented emergence of horse domestication across Eurasia, evidence for the presence, let alone consumption, of horse products in prehistoric Sicily remains absent or ambiguous. While horses transformed human mobility, warfare, and trade in many parts of Europe and the Near East [9–11], their role in insular Mediterranean societies, and specifically in Early Bronze Age Sicily, remains poorly understood.

Current models of Sicilian prehistory rely almost exclusively on indirect evidence and have long assumed that horses were absent from human diets or ritual economies during the third millennium BCE. Faunal remains are scarce and often compromised by poor stratigraphy or later contamination. Thus, no biomolecular evidence has yet confirmed the presence or consumption of equine products on the island during this period.

Turning to prehistoric Italy (Table 1), domestic horses are attested from the Eneolithic, often linked to ritual contexts [12]. However, until the Middle Bronze Age, they appear to have remained rare and symbolically charged rather than economically integrated. In Sicily, such remains are even more elusive, and the role of horses in indigenous cultural development has remained speculative until the Iron Age.

**Table 1:** Chronological chart of Italian prehistory with comparative reference to the different nomenclature and sequence system adopted for the Bronze and Iron Ages in continental Italy and Sicily.

Italian peninsula	Sicily
	**Late Epigravettian** 13000−9600 BCE
**Mesolithic** 11200–6000/5500 BCE	
**Neolithic** 5500-3800/3500 BCE	
**Copper Age** 3600-2300/2200 BCE	
**Early Bronze Age I-II** 2300/2200–1700 BCE	**Early Bronze Age** 2300–1550 BCE Castelluccio culture
**Middle Bronze Age** 1700–1350 BCE	
**Recent Bronze Age** 1350–1150 BCE	**Middle Bronze Age** 1550/1450–1250 BCE Thapsos culture
**Final Bronze Age** 1150–950 BCE	**Late Bronze Age** 1250–1050 BCE Pantalica Nord culture
**Early Iron Age** 950–750 BCE	**Early Iron Age** 1050–950 BCE Cassibile culture

To address this lacuna, the present study investigates whether horses were processed and consumed by Early Bronze Age communities in central Sicily, using direct biomolecular evidence recovered from ceramic residues. Drawing on combined lipidomic and proteomic analyses of pottery from the site of Polizzello Mountain (Mussomeli, Caltanissetta province), this research tests the hypothesis that equine-derived substances, especially blood and fat, were present in ritual or culinary contexts associated with the Castelluccio cultural horizon.

This study contributes to broader debates about equid domestication, human-animal relationships, and food practices in the prehistoric Mediterranean, while introducing new analytical data to challenge long-held assumptions about the invisibility of horses in early insular diets. It is part of the University of South Florida’s Mediterranean Diet Archaeology Project [13–23].

## 2. Archaeological context

### 2.1 Equids in early prehistoric artistic expressions

The island of Sicily has a well-known artistic record that has been usually dated to Late Epigravettian and Mesolithic based on its formal aspects and style. The first radiocarbon dates recently produced in direct connection with the artistic record seem to confirm this attribution [24]. The ca. 100 single figures with a figurative (i.e., not abstract) subject that are generally considered of this age [25] present characteristics that are in common with the coeval European record (animalistic subjects with a reduced number of species depicted) as well as other that seem to be highly exacerbated in the Sicilian (engraving as largely dominant, possibly unique technique, concentration of the depictions in a very limited number of sites, namely 3).

**Fig 1:**
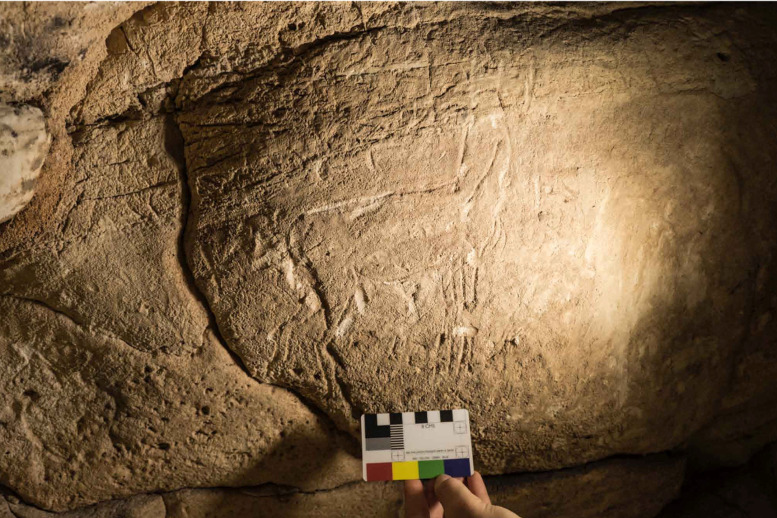
Characteristics of the equids’ representations in the artistic record of Late Glacial Sicily.

**Fig 2:**
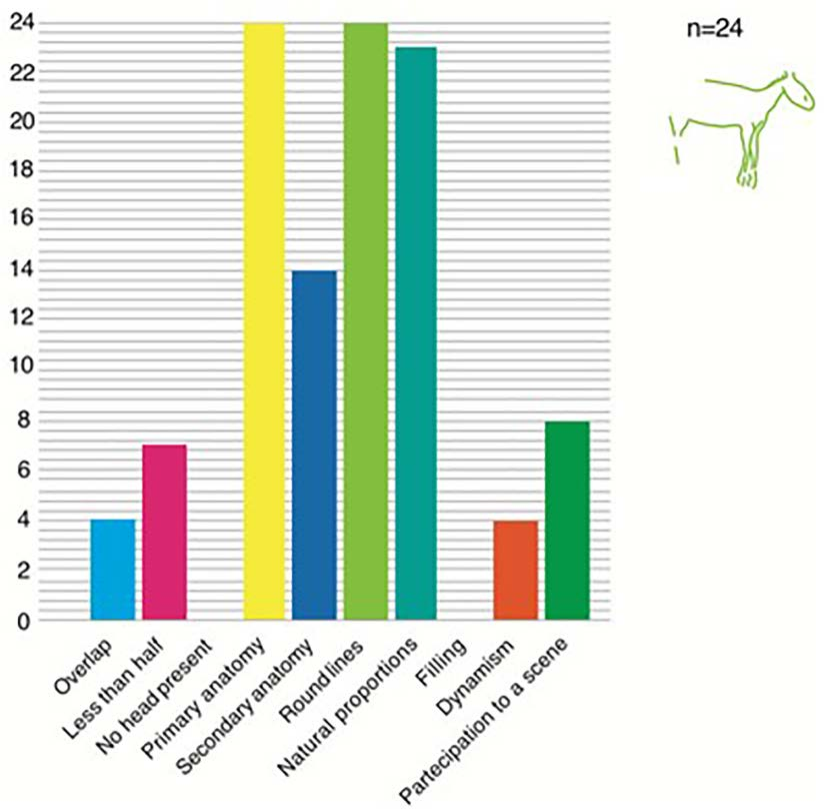
The engraved horse in Panel A at the Grotta del Genovese, Levanzo (photo author G. D. M.).

If we exclude the human figures (n = 21, they are almost all part of a single scene, the well-know one at the Addaura cave), the *Equidae* are the most frequent with 24 entries (Fig 1), followed by the *Bovidae* (with 18) and *Cervidae* with 11. Equids are also one of the only two species present outside the 3 caves where the art depictions concentrate (the other being cervids). Equids are represented with a relatively naturalistic style (Fig 2) and the constant use of rounded lines. All of them have primary anatomical traits (that allow a safe attribution to the class and often also secondary traits present (mouth, tail or tongue, ca. 58%). Thanks to the precision in their depiction, a few of the equids could also be assigned more specifically to a species, the now-extinct wild donkey (*Equus hydruntinus*). Like all other animals in the record (with the sole exception of the human figures, that seem indeed to constitute a class of their own, as already noted), equids are only limitedly depicted in a dynamic pose and mostly as a single figure (only 4 being part of scene).

This predominance of equids depiction in the Upper Paleolithic figurative art record of Europe has already known for quite some time ([26] with the sole exception of the Pyrenees where Bisons are the most represented species, while horses score anyway second). Sicily in this regard shows no differences with the Western European record [25].

That the animals depicted during the Upper Paleolithic represent a small fraction of the Pleistocene fauna, has been already abundantly discussed [27–29]. On the reasons why precisely equids, bovids and cervids are among the most represented species, no consensus has been reached yet, and it is likely that the reasons for such a predominance might indeed have changed along the course of several millennia of artistic production. There is very little doubt though, that these animals must have played an important role in the cosmological universe of the human groups that depicted them.

### 2.2 Zooarchaeological evidence for equids in Sicilian prehistory

In Sicily, both the horse and donkey are securely attested beginning in the Iron Age. Yet, Villari [30], in his review of numerous prehistoric Sicilian contexts, reports four equid remains (*Equus* sp. cfr. *caballus*) within the Neolithic faunal assemblage of Matrensa, as well as a fragment from a Neolithic level at Megara Hyblaea, possibly part of a tool handle, which he attributes to a donkey (*Equus asinus*). In addition, from Hut VI at the Castelluccian site of Monte Casale, Villari records two fragments attributed to the horse (*Equus caballus*). All these equid remains derive from rather early excavations conducted by Paolo Orsi at the beginning of the 20^th^ century [31–33], thus raising concerns regarding the chronological and stratigraphic reliability of these finds. Villari [30] further notes that all the sites show significant evidence of Greek-era stratigraphic overlaps. Such evidence may indicate the contamination of prehistoric layers with materials from historical periods, a problem likely stemming from excavation methodologies that, at the time, were not fully developed or organized according to rigorous stratigraphic criteria.

However, it should be emphasized that Sicily was already home to a wild equid species, *Equus hydruntinus*, from the Middle Upper Pleistocene onward. This species, which inhabited much of southern Europe during the Late Pleistocene and the Early Holocene, was first described by Regalia in 1907 based on remains from Grotta Romanelli in Apulia. *Equus hydruntinus* was characterized by a slender build and long, thin limbs suited for running over hard, arid terrain. Its dental morphology, featuring reduced incisors and molars, suggests a diet adapted to the herbaceous plants typical of steppe environments. The species was widely distributed throughout southern and eastern Europe, with finds ranging from Spain to Iran [34]. In Italy, principal evidence comes from Lazio, Abruzzo, Apulia, and especially Sicily, which appears to have served as one of the species’ last European refuges before its eventual extinction in the Late Holocene. Its presence on the island was facilitated by glacial periods that lowered sea levels and created land bridges between the Italian peninsula and Sicily. In particular, the lowered sea level during the second Würmian pleniglacial appears to have enabled its migration into Sicily, placing it among the faunas of the S. Teodoro-Pianetti Faunal Complex [35–38]. The subsequent arrival of continental faunas, known as the Castello Faunal Complex, in some sense marks the establishment of *Equus hydruntinus* in the island’s prehistoric environments and its interactions with the human communities inhabiting Sicily. In fact, unlike the scant remains identified in the preceding S. Teodoro-Pianetti Complex, numerous bones of this equid have been recovered from Epigravettian sites in association with lithic assemblages [39,40].

During the Mesolithic, sporadic occurrences are noted in the faunal assemblage from Grotta del Genovese on Levanzo [41]. Although these are limited, they gain interest when considered alongside rock engravings at the site that may depict *Equus hydruntinus*. However, the dating of these rock carvings remains unclear, and within Mesolithic layers, the remains of this animal are markedly less common than in the Pleistocene levels of Sicily [34]. In the subsequent Neolithic period, no remains clearly attributable to *Equus hydruntinus* have been identified, with a few exceptions reported from the Neolithic levels at Grotta delle Prazziche (Lecce, Apulia) [42] and Grotta delle Mura (Monopoli, Apulia) [43]. Regarding Sicily, there is no clear evidence of the species’ persistence into the Neolithic. Nonetheless, the equid fragment from Megara Hyblaea, initially identified as donkey, might raise intriguing possibilities regarding a prolonged Holocene survival of *Equus hydruntinus* or a possible premature introduction of the *Equus caballus*.

Such a scenario would warrant re-examination of the specimens using modern molecular and radiometric analytical tools, potentially resolving uncertainties in taxonomic attribution and chronological placement.

In Sicily, the habitat of *Equus hydruntinus* included grasslands and open landscapes that, while undergoing climatic transformations, remained suitable for its survival until the onset of the Holocene. The extinction of these equids in Sicily and the rest of Europe is attributed to a combination of climatic and anthropogenic factors. With the global warming that followed the end of the Last Glacial Maximum, rising sea levels isolated Sicily from the Italian peninsula, reducing the open spaces necessary for the species’ survival [44]. The expansion of forests and Mediterranean scrub further fragmented its habitat. In addition to these environmental pressures, it is likely that significant human hunting contributed to the species’ decline. *Equus hydruntinus* remains are frequently associated with lithic tools and butchery marks in archaeological sites, indicating that it was a regular prey item for Epigravettian hunters. Their activities appear to have accelerated the extinction of a species already weakened by environmental changes [34].

Nevertheless, thanks to its geographical position and environmental diversity, Sicily was probably one of the last European refuges of *Equus hydruntinus* [45]. Research continues to reveal new details about its biology and its interactions with prehistoric humans. Future discoveries and more advanced ancient DNA analyses may yield further insights into its evolution, survival, and the causes of its disappearance. In this regard, Sicily remains a fundamental reference point for studies in Mediterranean paleontology and zooarchaeology.

### 2.3 The case study of Polizzello Mountain

**Fig 3:**
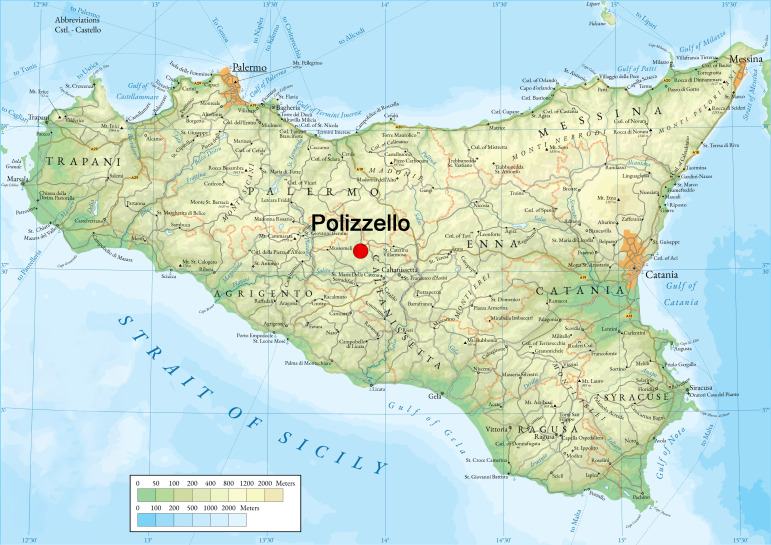
Map of Sicily with the location of Polizzello mountain (source Wikimedia Commons, CC BY 4.0, https://commons.wikimedia.org/wiki/File:Sicily_Map.png).

Polizzello Mountain, in central Sicily between Caltanissetta and Agrigento (Fig 3), is a key site for understanding Iron Age indigenous societies and their interaction with Greek settlers [16]. Continuously occupied from the mid-11^th^ to mid-5^th^ century BCE, its acropolis functioned as a major sanctuary. Strategically located with views over the Upper Platani Valley, it hosted communal rituals consistently evidenced over the course of several centuries by votive pits, animal sacrifices, and a large circular stone enclosures of rectangular and circular shape [46]. These held ritually broken ceramics, faunal remains, and elite votive items, reflecting cycles of feasting and sacrifice. Imported and local Greek-style artifacts further attest to intercultural exchange. After a brief hiatus, the site was reused in the 5^th^ century BCE for domestic purposes, losing its sacred role.

Evidence for an earlier occupation of the Polizzello Mountain emerged during the excavations directed by Paolo Orsi in 1926, when a megalithic chamber tomb was identified on the eastern slope of the mountain in the *Abbeveratoio* locality. Such type of tomb, rare but typical burial of the Early Bronze Age (EBA) and attested in a good number of sites of the Castelluccio culture, is traditionally associated with the emergence of local elites through the adoption of foreign funerary practices, in this case originated from the Maltese archipelago [47]. The exploration of this context, never published and only documented in the drawing field-book of Rosario Carta, who very likely conducted the fieldwork on behalf of Orsi [48], remained since then the only known EBA context at Polizzello.

**Fig 4:**
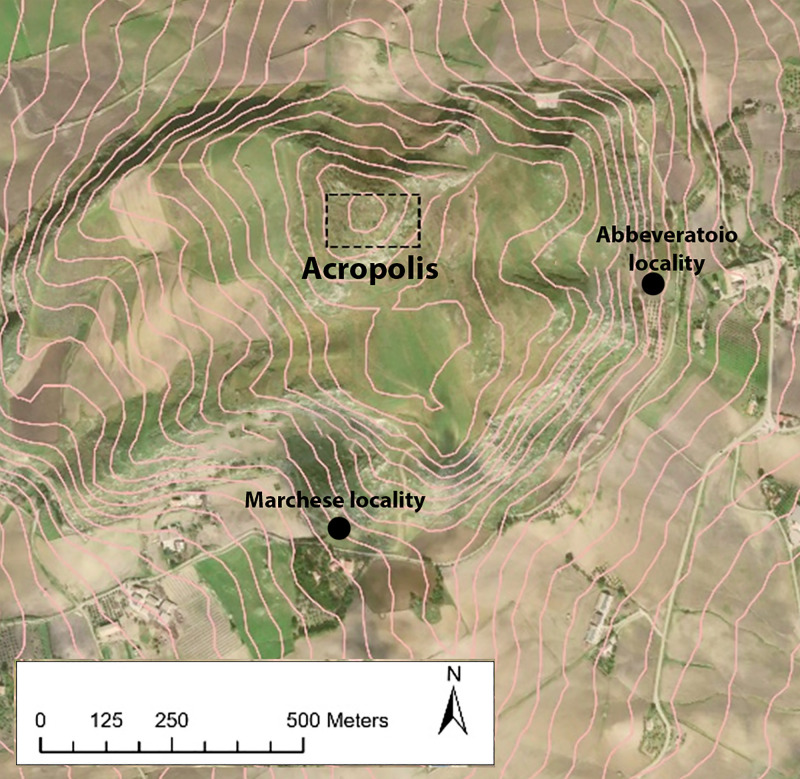
Map of Polizzello Mountain with the indication of the two EBA sites, at Abbeveratoio and Marchese localities (GIS elaboration from the author D.T., base image from NASA Earth Observatory, CC BY 4.0).

**Fig 5:**
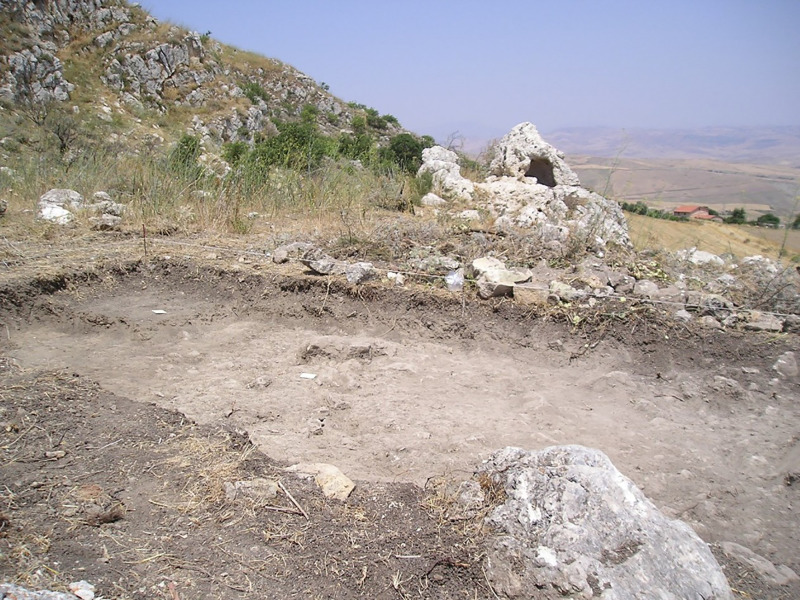
Stone enclosure at the beginning of the excavation, view from south-west, in the background the conical boulder (photo author D.T.).

In 2005, during a series of targeted archaeological excavations related to clearing a perimeter around the Polizzello Mountain for the construction of a permanent fencing system, in a small plateau on its southern slopes, a locality known as Predio Marchese or Marchese locality, a new Early Bronze Age site was identified (Fig 4). The site (LAT 37°36’7.09“ N; LONG 13°49’32.44” E) comprised two nearby areas, a stone enclosure and a boulder with a natural conical shape, with a niche excavated in it, 15 meters east of the former (Fig 5). The plateau presented in the topsoil a good presence of scattered Castelluccio pottery and, about 100 meters north of it, it was overlooked by two isolated chamber tombs of Castelluccio type excavated on the upper ridge of the southern flank and very likely already looted, both additional evidence for a more widespread occupation of this area in the EBA.

**Fig 6:**
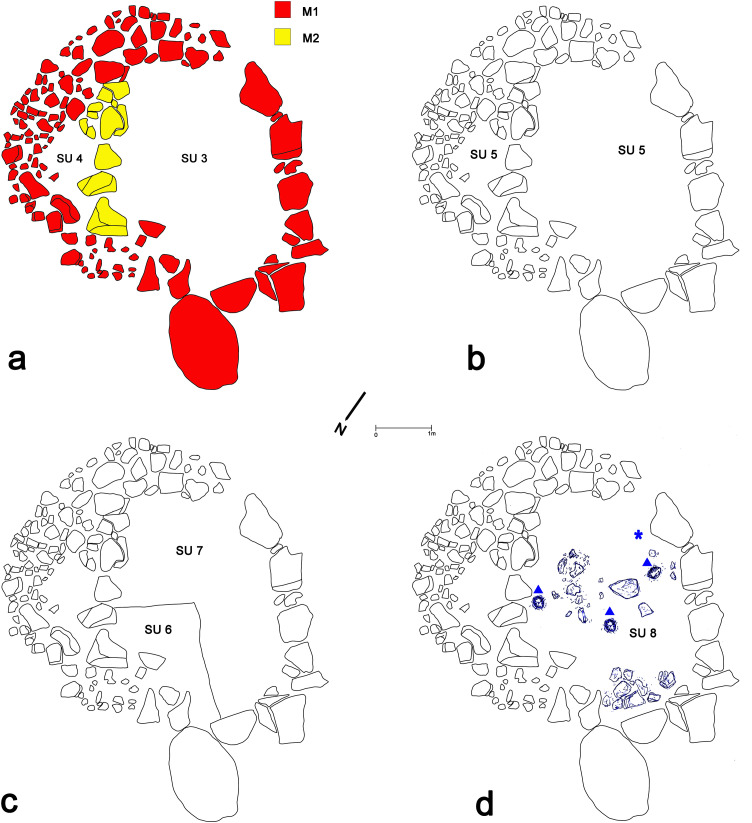
Main phases of uses of the stone enclosures with indication of the floor levels and structures M1 and M2 (drawings by M. Cocciadiferro, digital elaboration author D.T.).

After the removal of the first deposit below the topsoil (SU 1), the excavation of the stone enclosure started out in a trench of 6 x 6 m at a depth of −0.65 m (0 = 771.46 m a.s.l.) with the identification and removal of a context of soil (SU 2) marking the abandonment of the structure (Table 2). The excavation revealed that the stone circle had a diameter of 4.6 m, and entrance of 0.5 m of width on the southern side, that its internal space was separated by a septum subsequently added on the eastern side (M1-M2). Below SU 2, it was identified a hard-packed dirt floor level, SU3–4, in the entire internal area of the structure (Fig 6a). Right underneath it, an older similar level, SU 5, was uncovered (Fig 6b). Below SU 5, it was identified a deposit of two similar layers, SU 6 and 7, characterized by abundance traces of charcoal and ashes, possibly documented a destructive activity (Fig 6c). Below this deposit, an additional floor level of the same type, SU 8, was uncovered, revealing on it three shallow pits of circular shapes of about 0.20 m of diameter each (Fig 6d).

**Table 2:** Summary of the contexts identified during the excavation of the stone enclosure marking major phases in connection with absolute dates.

Context	Heights	Type	Phases	^14^C Dates
SU 1	−0.40/0.50 m	Deposit	–	–
SU 2	−0.65 m	Deposit	Abandonment	3700 ± 25 (^14^C age), 2196−1993 BCE (2 σ range) *Capra* second upper right molar
SU 3–4	−0.80/-0.83 m	Floor level	Installation of the 3^rd^ floor level	–
SU 5	−0.90 m	Floor level	Installation of the 2^nd^ floor level	3760 ± 20 (^14^C age), 2281−2085 BCE (2 σ range) *Capra* second upper left molar
SU 6	−1.10 m	Deposit	Destruction/fire	3780 ± 20 (^14^C age), 2287−2139 BCE (2 σ range) *Bos* second lower left molar
SU 7	−1.35 m	Deposit	Destruction/fire	–
SU 8	−1.40/-1.45 m	Floor level	Installation of the 1^st^ floor level	–

The retrieval of animal bone specimens in SU 2, SU 5, and SU 6 allowed us to obtain radiocarbon data that proved how the site falls well inside the traditional chronology of the Castelluccio culture. For the four samples, their collagen yields were sufficient and produced reliable C and N isotope results. The collagen sample was combusted at 575 °C in evacuated/ sealed ampoules in the presence of CuO. The resulting carbon dioxide was cryogenically purified from the other reaction products and catalytically converted to graphite using the method of Vogel et al. [49]. Graphite ^14^C/^13^C ratios were measured using the CAIS 0.5 MeV accelerator mass spectrometer. The sample ratios were compared to the ratio measured from the Oxalic Acid I (NBS SRM 4990).

The sample ^13^C/^12^C ratios were measured separately using a stable isotope ratio mass spectrometer and expressed as δ^13^C with respect to PDB, with an error of less than 0.1‰. The quoted uncalibrated dates have been given in radiocarbon years before 1950 (years BP), using the ^14^C half-life of 5568 years. The error is quoted as one standard deviation and reflects both statistical and experimental errors. The date has been corrected for isotope fractionation. The calibration was done using the Calib Rev 8.1.0. program with the IntCal20 calibration dataset [50].

**Fig 7:**
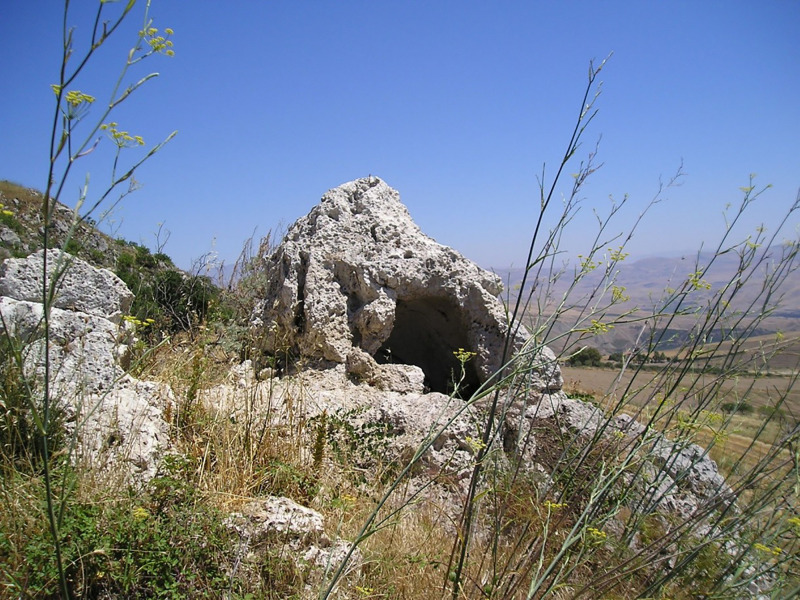
Conical boulder with the niche, from the West (photo author D.T.).

**Fig 8:**
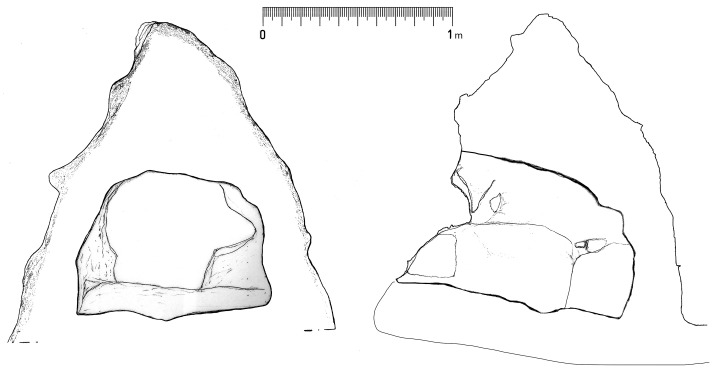
Front view and section views of the conical boulder with the niche (drawings by M. Cocciadiferro).

The massive conical boulder, rolled from the upper slope of the Mountain, was in a scenic position at the southern edge of the plateau before a jump of about 20 meters. On its western side, a rectangular niche of 1.30 m (height) x 1.00 m (width) x 1.15 m (depth) was excavated with internal surfaces intentionally left rough and without any sign of closing system (Figs 7 and 8). In it, there was just one deposit of soil mixed with pottery fragment, SU 9.

**Fig 9:**
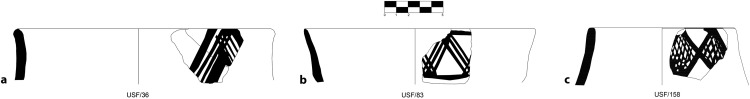
Castelluccian open shapes. a-b) Bowls USF/36 and USF/83 decorated with series of repeated and overlapping elements namely from SU 2 and SU 4 c) Jar USF/158 decorated with mirrored designs from SU 5 (drawings by F. Lo Faro).

**Fig 10:**
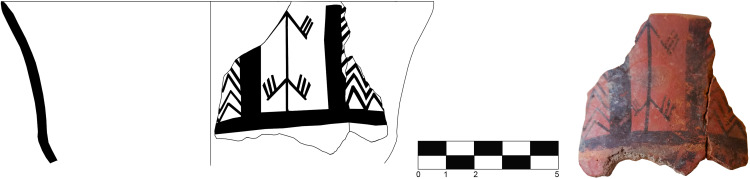
Castelluccian bowl USF/128 decorated with vertical sequences of repeated elements within metopes framed by solid bands and schematic representation of a human figure from SU 2 (drawings by F. Lo Faro, photo author D.T.).

**Fig 11:**
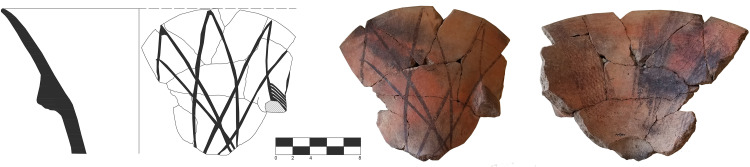
Castelluccian pedestal basin USF/110 found in the niche (SU 9) decorated with reticulated bands decorating its interiors (drawings by F. Lo Faro, photo author D.T.).

**Fig 12:**
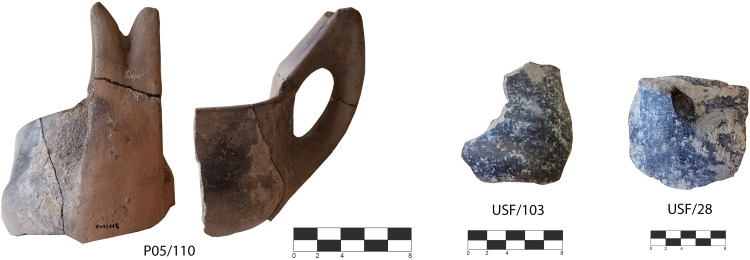
Rodi-Tindari-Vallelunga dipper cups. P05/110 from SU 1, USF/103 from SU 6 and USF/28 found in the niche (SU 9) (photo author D.T.).

With respect to the materials retrieved in the stone circle and in the niche the vast majority are pottery fragments consisting of medium- and large-sized fragments of cooking ware and table ware, fitting seamlessly into the Castelluccio culture due to its recognizable shapes and decorative motifs, which are well-documented in the region. The decorative motifs include recurring patterns [51] on open shapes such as series of repeated and overlapping elements (Fig 9a-b) and mirrored designs (Fig 9c) as well as vertical sequences of repeated elements within metopes framed by solid bands [52], in certain rare instances, such as this one, portraying schematic representations of human figures (Fig 10). Reticulated bands decorating the interiors of footed vessels, like in the case of the exquisite example of pedestal basin found in the niche (Fig 11), find precise parallels at Muculufa [53]. Additionally, parallel vertical or rhomboidal lines and horizontal patterns on vessel handles and feet ([54], tav. IV, 9). In terms of shapes, the assemblage includes footed vessels, pitchers, and cups: objects typical of libation rituals. The typology of the finds from Polizzello shows clear parallels with Castelluccian ceramics from both geographically proximate sites and others across Sicily. Of particular interest is a gray ceramic cup with burnished surfaces and a raised handle terminating in two pointed ends (Fig 12) that belongs to the production known as Rodì-Tindari-Vallelunga (RTV), alternatively interpreted as a conservative comeback of an older regional pottery style [55] or as new pottery styles developed under the influence of foreign cultures with ties in Southern Italy [56]. The coexistence of Castelluccio footed vessels and RTV dipper cups has numerous exact parallels [57]. This association is also attested in another significant EBA votive context at Ciavolaro di Ribera (Agrigento). As argued elsewhere, the presence of RTV dipper cups in tandem with Castelluccian containers likely represents a Castelluccian practice of enriching their ritual inventory with an “exotic” object [55].

**Fig 13:**
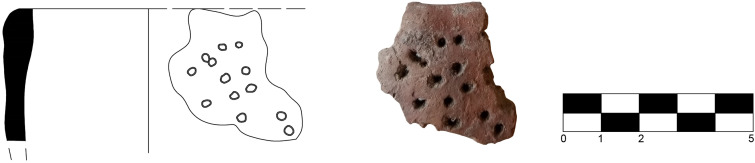
Strainer vessel P05/135 from SU 2 (drawings by F. Lo Faro, photo author D.T.).

Among the ceramic materials, it is noteworthy the identification of fragments of a coarse ware strainer vessel with cylindrical body (Fig 13) traditionally associated in Sicilian prehistoric contexts with dairy making activities, a theory [58,59] recently confirmed by proteomics analyses [19].

**Fig 14:**
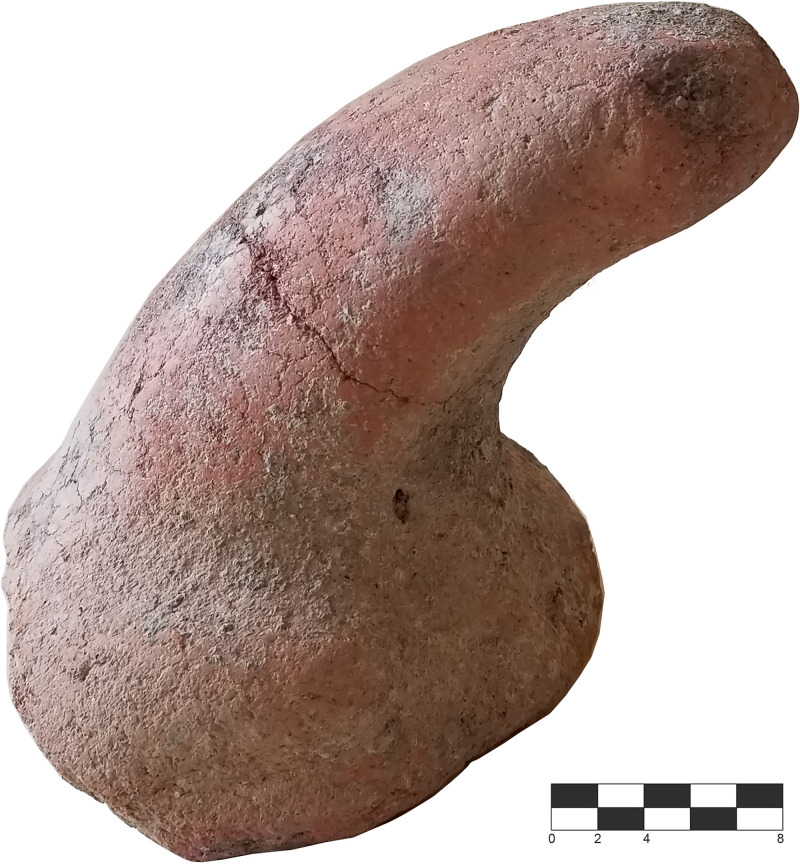
Large terracotta phallus from SU 8 (photo author D.T.).

Besides the pottery, noteworthy is the discovery of a large terracotta phallus, found in situ on the older floor level identified (SU 8), between the entrance and one of the pits (marked with a “*” in Fig 6d, Fig 14). Such artifacts connected with fertility rituals among of the exclusive indicators of the Castelluccio culture [56,60]. Additionally, three stones for grinding and smoothing were recovered on the floor levels of the stone circle alongside a good number of flint flakes and two fragments of flint blades. The evidence for animal skeletal remains is extremely limited and represented by a few teeth and two fragments of long bones overall attributable to *Capra*, *Bos* and *Sus*.

From an archaeological perspective, the two main features identified in this context, the stone enclosure and the boulder with the niche, do not immediately appear to be classifiable as for domestic or funerary use. The enclosure was not a dwelling, lacking any form of elevation and coverage. The boulder containing the niche is several hundreds of meters farther from the rocky slope of the Mountain, where actual Early Bronze Age chambers tombs looted in antiquity as still visible, and the niche itself lacks sufficient space for a burial and features related with a closing system (frame and hinges). Therefore, it is likely that the two spaces were used for repeated performances involving food preparation and consumption (stone enclosure), offering (the niche in the boulder), possibly in the frame of a ritual connected with fertility as suggested by the discovery of the terracotta phallus.

In order to better understand the essence of the activities conducted in the stone circle and the relationship between such activities and the evidence offered by the niche in the conical boulder, 54 pottery samples were extracted from Castelluccio and RTV ceramic vessels, from all the stratigraphic units identified, and submitted to organic residues analyses via Gas-Chromatography Mass Spectrometry and Proteomics Mass Spectrometry.

## 3. Materials

A total of 54 samples were collected for organic residue analysis from a large assemblage of fragmented Castelluccio pottery retrieved on the floor levels of the stone enclosure and the niche and the area immediately in front of it. During the selection of the samples, shapes typologically diagnostic and representing both fine and coarse ware were considered.

## 4. Methods

From the interior surface of each potsherd, residues were collected by the grattage method (scraping with a clean scalpel). The first layer was discarded to minimize contamination. The scraped material was immediately ground to a fine powder using an ethanol-sterilized mortar and pestle. To prevent cross-contamination, all equipment was thoroughly cleaned between samples with 95% ethanol and Milli-Q water. A total of 54 samples were obtained from ceramic fragments, each placed in a sterile 1.5 mL microcentrifuge tube and labeled with a unique code corresponding to the artifact’s context. To facilitate a single-blind analysis, archaeological sample codes were replaced by sequential numerical identifiers (1–54) recorded in a lookup table. Among these, 25 samples were selected for proteomic analysis based on context and initial screening, while all 54 were subjected to lipid residue analysis. Sample aliquots for different analyses were handled in parallel to ensure representative splits for lipidomics and proteomics.

### 3.1 Lipid Extraction and GC-MS Analysis

Lipid residues were extracted from the pottery powders using a solvent-based protocol optimized for ancient lipid recovery. Approximately ~0.5–1.0 g of each powdered sample was combined with 1 mL of *n*-heptane (HPLC grade) in its tube. Tubes were sealed with Parafilm and placed in an ultrasonic bath for a total of 90 minutes, with 15-minute rest intervals every 30 minutes to prevent overheating. Sonication in heptane facilitates the dissolution of lipids absorbed in the ceramic matrix. After sonication, 0.5 mL of 2 M methanolic potassium hydroxide (KOH in methanol) was added to each sample. This transesterification step derivatizes free fatty acids and glycerolipids into fatty acid methyl esters (FAMEs), increasing their volatility for gas chromatography. The tightly capped tubes were vortexed vigorously for ~2 minutes to ensure thorough mixing of the alkaline methanol with the heptane extract. The derivatization (base-catalyzed methylation) converts fatty acids to their methyl esters by masking polar carboxyl groups, thereby improving their GC amenability.

Due to limited sample availability and the need to minimize destruction of valuable archaeological material, a non-polar solvent (n-heptane) was selected for lipid extraction. We acknowledge that this approach may bias against recovery of polar lipids such as phospholipids and sterols. This limitation is recognized and addressed in the discussion as a direction for future research.

Following derivatization, the samples were centrifuged at 12,000 × g for 10 minutes to separate the organic (heptane) phase from the methanolic KOH phase. Tubes were counterbalanced with water-filled tubes to equalize mass in the rotor. Immediately after centrifugation, the upper organic layer (containing heptane and dissolved lipid derivatives) was carefully pipetted off (0.8 mL). Care was taken to avoid disturbing the lower aqueous phase or any interfacial debris, since carryover of the alkaline solution could damage the GC-MS column. The heptane extracts were transferred to amber GC vials, sealed with Teflon-lined caps, and labeled with the sample ID. The extracts were stored at 4 °C until analysis. Procedural blanks (solvent and reagents without sample powder) were processed alongside the ancient samples to monitor laboratory contaminants. Although silylation using agents such as BSTFA could have improved detection of sterols and tocols, the available sample mass was insufficient to permit a second derivatization step.

GC-MS analyses were performed on an Agilent 6890 gas chromatograph coupled to a 5973 Inert mass selective detector (MSD) operated in electron ionization (EI) mode. The instrument was equipped with an autosampler (Gerstel MPS2) and a DB-5ms UI capillary column (30 m × 0.25 mm i.d., 0.25 μm film thickness). High-purity helium was used as the carrier gas at a constant flow rate (1 mL/min). One microliter of each heptane extract was injected in splitless mode at an injector temperature of 300 °C. The GC oven was programmed from 55 °C (initial hold 1 min) to 150 °C at 10 °C/min, then ramped to 300 °C at 15 °C/min, with a final hold of 5 min at 300 °C. This temperature program allows efficient elution of compounds ranging from short-chain FAMEs to long-chain saturated hydrocarbons. The MSD scanned *m/z* 35–450 in full scan mode. Mass spectral data were acquired and processed with Agilent ChemStation software. Compounds were identified by comparison of their mass spectra and retention times to reference libraries and standards: NIST 05 mass spectral library, a certified FAME mix (C8–C24 FAMEs, Supelco), and an *n*-alkane standard mixture (C21–C40, Sigma-Aldrich). Matches were accepted based on good spectral library fit (>90%) and alignment with expected retention indices (as calibrated by the *n*-alkane series). Key lipid components of interest were the saturated and unsaturated fatty acid methyl esters, as these can indicate the presence of animal fats or plant oils in archaeological residues.

We specifically screened for molecular biomarkers such as sterols or terpenoids (e.g., cholesterol for animal fats, or plant sterols like stigmasterol for plant oils) to gain source information. However, given the extraction and derivatization protocol (alkaline methanolysis without silylation), free sterols would not be derivatized and thus were expected to have low detectability. Nonetheless, any sterol peaks observed were noted. All samples were analyzed in random order, interspersed with solvent blanks and standard mixtures to monitor for carryover and instrument stability. After each batch of ~12 injections, the column was cleaned with multiple blank runs to flush any residues. No appreciable lipid carryover was detected in blank runs. Because of sample scarcity and the need to preserve as much material as possible, internal standards were not used. Consequently, all lipid results are qualitative and interpreted in terms of presence/absence and intra-sample comparison rather than quantitative concentration.

### 3.2 Proteomic Extraction and LC-MS/MS Analysis

Selection of 25 sherd samples was based on vessel typology (e.g., strainers, basins, and ritual cups), stratigraphic integrity, and the archaeological context suggesting culinary or ceremonial use. Preliminary lipid results also informed selection, favoring samples with observable organic content and strong preservation indicators. Approximately 30–200 mg of ceramic powder from each selected sherd was used for protein extraction, employing a two-step sequential extraction to maximize protein yield. Each powder sample was split into two equal aliquots (~15–100 mg each). The first aliquot was extracted under acidic conditions (pH ~ 2) by adding 4% (w/v) sodium dodecyl sulfate (SDS) in 0.1% trifluoroacetic acid (TFA). The sample in acidic SDS buffer was incubated at 95 °C for 30 min to solubilize acid-soluble and loosely bound proteins. The second aliquot was extracted under alkaline reducing conditions (pH ~ 8.5) by adding 4% SDS in 100 mM Tris-HCl with 0.1 M dithiothreitol (DTT). This sample was likewise heated at 95 °C for 30 min, targeting proteins that extract better at high pH with a reducing agent (to break disulfide bonds). The volume of extraction buffer added was five times the sample mass (e.g., 500 μL buffer for 100 mg powder) to ensure a high buffer-to-sample ratio. After cooling, the two extracts for each sample were pooled to combine the complementary protein fractions. Insoluble debris was removed by centrifugation (10 min at 14,000 × g), and the supernatant containing extracted proteins was carefully recovered.

The protein extracts were then purified to remove soil-derived inhibitors and humic contaminants. A commercial precipitation and clean-up kit (PlusOne 2-D Clean-Up, GE Healthcare) was used according to the manufacturer’s protocol. This step precipitates proteins while removing SDS, salts, and other non-protein contaminants. The resulting protein pellets were air-dried and re-dissolved in 100 μL of 50 mM ammonium bicarbonate buffer (pH 8.3) containing 0.1 M DTT. The samples were incubated at room temperature for 3 h to allow DTT to reduce cystine disulfide bonds, fully denaturing the proteins. Protein concentration of each extract was measured with a Qubit™ fluorometric protein assay (Thermo Fisher Scientific) to estimate yield. Following reduction, cysteine residues were alkylated by adding iodoacetamide (IAA) to a final concentration of 0.2 M and incubating for 1 h in the dark at 20 °C. Alkylation prevents re-formation of disulfide bonds and marks cysteine sites with carbamidomethyl groups (+57 Da), aiding peptide identification. Each extract was then enzymatically digested with trypsin (Thermo Fisher Sequencing-Grade Modified Porcine Trypsin) to cleave proteins into peptides. Trypsin was added at an enzyme-to-protein ratio of 1:50 (w/w), and digestion was carried out overnight (~16 h) at 37 °C. The digestion was quenched by acidification (addition of formic acid to ~1% final). The resulting peptide mixtures were concentrated to dryness in a vacuum centrifuge and reconstituted in 50 μL of 5% aqueous formic acid. The reconstituted peptide solution was centrifuged through a 0.2 μm spin filter to remove any remaining particulates.

Peptide analysis was performed by nano-scale liquid chromatography tandem mass spectrometry (nanoLC-MS/MS) using a Vanquish Neo UHPLC system (Thermo Scientific) coupled to a high-resolution Orbitrap Exploris 240 mass spectrometer (Thermo Fisher Scientific). For each sample, 1 μL of the peptide solution (typically containing ~0.1–1 μg of peptides) was injected. Peptides were first loaded onto a C18 trap column (Thermo Acclaim PepMap 100, 100 μm × 2 cm, 5 μm particles) at 7 μL/min in 100% solvent A (0.1% formic acid in H₂O) for 3 min. The trap was then switched in-line with the analytical C18 column (Thermo PepMap RSLC C18 EASY-Spray, 75 μm × 50 cm, 2 μm, 100 Å) maintained at 40 °C. Peptides were separated by an acetonitrile (ACN) gradient at a flow rate of 250 nL/min. The LC gradient was: 5% solvent B (0.1% formic acid in ACN) for 3 min, then 5% to 65% B over 85 min, then 65% to 95% B over 5 min, hold at 95% B for 5 min, then return to 5% B over 10 min and re-equilibrate for 15 min. This 120-minute method effectively resolves a complex peptide mixture. The mass spectrometer was equipped with a nano-ESI source (Thermo Scientific) and operated in positive ion mode. Peptide ions were generated by electrospray at 1.75 kV with the ion transfer tube at 275 °C.

The Orbitrap was operated in data-dependent acquisition mode to automatically sequence peptides. Full MS survey scans (m/z 200–1600) were collected in the Orbitrap at 120,000 resolving power (at m/z 200). The most intense peptide ions (charge states 2–4, intensity >1 × 10^3^) were selected for MS/MS fragmentation by high-energy collisional dissociation (HCD) with normalized collision energy of 35%. Fragment ion spectra were acquired in the linear ion trap in rapid scan mode (low resolution MS/MS) to maximize speed. The quadrupole isolation width for precursor ions was 1.6 Da to select a narrow m/z window around each target. A dynamic exclusion of 60 s was applied to minimize repeated sequencing of the same peptide; precursors were excluded from reselection within ±10 ppm of the selected m/z for 60 s. Monoisotopic precursor selection was enabled to prioritize the monoisotopic peak. The instrument was run in “top speed” mode with a 3 s cycle time, meaning as many MS/MS scans as possible were performed within 3 s after each survey scan. The parallelizable time feature was activated to utilize the full time of each Orbitrap scan for overlapping MS/MS acquisitions. External mass calibration was performed using a standard calibration mix (Pierce LTQ ESI Positive Ion Calibration Solution) before analyses. Raw data were acquired with Thermo XCalibur (v3.0) software.

### 3.3 Proteomic Data Analysis and Quality Control

The MS/MS data were searched for peptide identification using PEAKS Studio X+ (Bioinformatics Solutions) and further validated with common proteomics criteria. MS/MS spectra were matched against the UniProt SwissProt protein database (downloaded 2023) with taxonomy unrestricted (no species filter) to allow detection of any organism’s proteins. To evaluate potential over-assignment, we conducted parallel searches restricted to Vertebrata and Mammalia subsets of the SwissProt database. All high-confidence identifications of Equus albumin remained valid under these taxonomic constraints, supporting the robustness of the assignments as the lowest common ancestor. Search parameters allowed for variable modifications commonly observed in ancient proteins, including oxidation of methionine (+16 Da) and deamidation of asparagine/glutamine (+1 Da, + 0.984 Da). Enzyme specificity was set to trypsin (cleavage C-terminal to K/R, except next to P) with up to 2 missed cleavages allowed. Carbamidomethylation of cysteine (+57 Da) was set as a fixed modification (from IAA alkylation). A precursor mass tolerance of 10 ppm and fragment mass tolerance of 0.6 Da were used for matching (appropriate for Orbitrap MS^1^ and ion trap MS^2^). Peptide-spectrum matches were filtered to a 1% false discovery rate (FDR) using a target-decoy approach. Protein identification criteria followed accepted standards in paleoproteomics: a protein was considered confidently identified if at least two unique peptides (non-overlapping in the protein sequence) were matched at 1% FDR, and the protein was not detected in procedural blanks. We further required that the protein’s identified peptide sequences exhibit post-translational modifications consistent with ancient diagenesis (e.g., high extent of deamidation in Asn/Gln), to help distinguish ancient endogenous peptides from modern contaminants. All peptide sequences from archaeological samples were manually inspected for unexpected modifications or spectral anomalies. In practice, the dominant protein identified (see Results) was supported by many unique peptides, exceeding the minimum identification criteria by a wide margin, which provides high confidence in its authenticity.

Extensive contamination control measures were implemented throughout the proteomic workflow to ensure the validity of ancient protein identifications. A laboratory blank extraction (using a fired clay fragment with no expected protein, or an empty tube) was carried through the entire procedure of extraction, digestion, in parallel with analysis of the samples. LC-MS/MS analysis of this blank yielded only a few protein hits, all common lab contaminants (e.g., keratins, trypsin, etc.), indicating minimal background carryover. Ancient ceramic samples were processed in batches separate from any modern reference samples to avoid cross-contamination. The LC-MS autosampler and column were rigorously rinsed between runs: 3–5 blank injections (solvent only) were performed between each archaeological sample run. The final blank run in each series was also searched against SwissProt to verify that no sample-derived proteins were carried over. These precautions align with emerging standards for protein authentication in archaeological materials [61], helping to ensure that reported protein identifications represent genuine ancient residues rather than modern contaminants.

To monitor contamination across all stages of the analysis, we included procedural blanks processed in parallel with archaeological samples. These included fired, protein-free ceramic fragments subjected to the full extraction, digestion, and LC-MS/MS workflow. Additionally, single-use sterile tools, ethanol-sterilized mortars, and filtered pipette tips were employed throughout. Sample processing was conducted in batches spatially and temporally separated from any reference materials. LC-MS autosampler and columns were rigorously rinsed between injections with multiple blank runs, and negative controls were analyzed via the same database search protocol. Raw proteomic data, including all blank runs, have been deposited in ProteomeXchange under reference number PXD066335.

## 4. Results

The proteomic analysis of the organic residues revealed a clear biomolecular signature of horse (genus *Equus*) products in a substantial subset of the vessels. The most prominent identification was equine serum albumin (ALBU_HORSE, UniProt P35747), detected in 23 out of 54 sampled sherds. Albumin, a major blood protein, was identified with high confidence based on multiple unique peptides in each positive sample. Table 3 summarizes the proteomic identifications of albumin in the residues. Ten samples yielded especially robust evidence, each with ≥2 unique peptides matching horse albumin (in some cases up to 6–7 unique peptides; sequence coverage up to ~22%) – far above common identification thresholds (typically 2 unique peptides minimum for ancient proteins [61]). In these samples, the albumin peptides collectively cover diverse regions of the protein sequence, confirming the presence of the protein rather than sporadic peptide noise.

**Table 3:** Proteomic identification of horse albumin (ALBU_HORSE) in EBA Sicilian pottery samples. Each sample is listed with the number of total and unique peptides matching equine albumin and the sequence coverage achieved. All listed samples have ≥2 unique peptides, indicating confident identification.

SU	Inv. no	Sample no.	Proteins	UniProt ID	Lowest common ancestor	Coverage (%)	Peptides	Unique peptides	Deamidation (%)
**SU 9**	P05/110	1	ALBU_HORSE	P35747	*Equus*	22.1	9	7	30.9
**SU 1**	USF 28	8	ALBU_HORSE	P35747	*Equus*	20.3	10	7	29.8
**SU 1**	USF 44	14	ALBU_HORSE	P35747	*Equus*	13.0	6	3	23.3
**SU 2**	USF 48	15	ALBU_HORSE	P35747	*Equus*	14.8	7	3	26.5
**SU 6**	USF 103	42	ALBU_HORSE	P35747	*Equus*	12.7	5	2	31.7
**SU 7**	USF 104	43	ALBU_HORSE	P35747	*Equus*	20.9	9	6	28.1
**SU 7**	USF 105	44	ALBU_HORSE	P35747	*Equus*	6.9	3	2	26.6
**SU 7**	USF 109	46	ALBU_HORSE	P35747	*Equus*	6.9	3	2	24.3
**SU 7**	USF 119	52	ALBU_HORSE	P35747	*Equus*	19.9	9	6	32.1
**US 7**	USF 121	54	ALBU_HORSE	P35747	*Equus*	7.6	4	4	21.4

In all cases, the lowest common ancestor (LCA) of the identified albumin peptides was the genus *Equus*, which includes horses and donkeys. Because the tryptic peptides of serum albumin are highly conserved between horse (*Equus caballus*) and donkey (*Equus asinus*), we technically attribute the identification to “*Equus* sp. Albumin”. However, given the archaeological and zooarchaeological context, horses are the most likely source of these albumin residues in Bronze Age Sicilian pottery. Donkeys were present in antiquity but were primarily used as pack animals; their intentional slaughter or cooking is less attested. In contrast, horses, while socially valued, could be hunted or consumed especially in earlier periods before strict taboos developed [62]. Thus, for discussion we refer to the albumin as deriving from horse blood/meat. Importantly, equine albumin is not a common lab contaminant in the cRAP list (unlike bovine serum albumin, which is ubiquitous in laboratory reagents). The detection of *Equus* albumin across many samples – including multiple peptides unique to the *Equus* sequence and absent in other species – underscores that this is an authentic ancient signal rather than contamination. Moreover, the peptides showed extensive deamidation (Asn/Gln → Asp/Glu), between 21 and 32%, consistent with ancient protein diagenesis and further supporting their antiquity [19,63–65].

The albumin identified is a blood protein, suggesting that the residues in these vessels are likely derived from animal blood or flesh fluids. In cooked or stored meat, albumin from muscle blood can bind to the vessel’s interior. The detection of seven unique albumin peptides in the strongest cases (e.g., sample 1, with 22% coverage) provides unambiguous evidence that horse blood or meat was processed in those pots.

## 5. Discussion

The findings offered by our exercise constitutes the first biomolecular confirmation of horse product usage in prehistoric Sicily. Previously, the presence of horses in EBA diets was only hypothesized indirectly, since wild horses may have roamed Sicily and could have been hunted. Faunal remains of horses are extremely scarce in contemporary Sicilian sites, and in broader Bronze Age contexts horses are often rare in domestic refuse, presumably because they were not commonly slaughtered for food.

The ritual use of horse products within the elevated and symbolically charged landscape of Polizzello Mountain may be better understood through a broader comparative lens that considers the cosmological significance of the horse across ancient cultures. In numerous Indo-European traditions, the horse occupies a liminal role as a boundary-crosser between the realms of the living and the dead, the earthly and the divine. Whether pulling the solar chariot across the sky or accompanying the soul into the afterlife, the horse often functions as a sacred intermediary and ideal totemic animal due to its strength, speed, and directional symbolism [66–68]. In Tibetan cosmologies, similarly, the horse is closely tied to death, rebirth, and transformation, embodying transition across metaphysical spaces [69]. That this activity occurred at a mountain sanctuary, a location widely recognized cross-culturally as a liminal and elevated threshold between worlds, further supports the notion that the consumption and offering of equine substances in Early Bronze Age Sicily may have held ritual or cosmological significance, rather than being purely subsistence-driven. The convergence of high-altitude setting, specialized architecture, and horse-derived residues aligns with a broader Eurasian ritual logic in which horses were embedded within sacred cycles of death, fertility, and regeneration.

While the ceremonial function of horse consumption appears primary in this context, it is worth noting that horse meat, blood, and especially marrow possess favorable nutritional profiles. Compared to other ungulates such as Bos, horse meat is low in cholesterol and rich in water-soluble vitamins and essential fatty acids, including linoleic and α-linolenic acids, particularly concentrated in bone marrow [70–72]. Such attributes may have contributed to its selective use in ritual contexts.

Our proteomic results thus fill an important gap demonstrating that horses may have been utilized as a food resource by Early Bronze Age communities in Sicily. This aligns with a broader pattern emerging in later prehistory – for instance, in northern Italy’s Late Bronze Age, butchery marks on horse bones at Bovolone (Verona) indicate that horses were occasionally butchered and consumed despite their rarity in faunal assemblages [62].

Interestingly, aside from equine albumin, no other animal proteins (e.g., milk or muscle proteins from other livestock) were confidently detected in the proteomic dataset. Even the sample extracted from strainer vessel P05/135, traditionally associated with dairy production activities, did not produce any results. This contrasts with some other studies of prehistoric ceramics, where a wider suite of food proteins (from cereals, legumes, milk whey, muscle, blood, etc.) have been recovered. In a pioneering study of Neolithic Anatolian pottery, Hendy *et al.* identified dairy proteins from cattle/goat milk and blood proteins (hemoglobin) from multiple taxa (cattle, sheep/goat, and even deer) in different vessels [63]. The absence of non-equine proteins in our samples could be due to several factors: the selection of sherds (we targeted those that were protein-rich by prior knowledge and high unique peptide ratio), the possibly short-use or single-use nature of these vessels (maybe primarily for one type of food), or greater degradation of other proteins that were present in lower abundance. Albumin is a relatively small, soluble, and abundant protein in blood; it may survive diagenesis better or be more easily extractable from porous ceramics compared to larger, less soluble muscle proteins [61]. Additionally, our stringent identification criteria may have filtered out other proteins present at trace levels or with few peptides. It is possible that other animal or plant proteins were present but fell below confident detection. Regardless, the strong and repeated identification of equine albumin stands out as the primary proteomic signal, pointing to horse-derived contents. In any case, the mere presence of a such a rare and ultra-specialized shape like P05/135, chemically ascertained in other studies as tied with cheese making activities (the strainer separated the curds from the whey) proves that this type of food stuff was produced and eventually consumed on site.

Our proteomic findings gain added credibility when considered alongside the lipid residue evidence, even though the latter on its own is not species-specific. All 54 pottery samples yielded extractable lipid residues, indicating these vessels had preserved organic matter (fats/oils) despite ~4000 years of burial. GC-MS analysis identified a suite of fatty acids and related compounds in the residues (Table 4). The lipid profiles were generally dominated by long-chain fatty acids: the saturated C16:0 (palmitic) and C18:0 (stearic) acids were among the most common residues (detected as their methyl ester derivatives), as inferred from the frequent presence of *n*-hexadecane (C16 aliphatic chain, from palmitic acid) and *n*-octadecane (C18 chain, from stearic or other C18 acids) in the chromatograms. Monounsaturated C18:1 fatty acid (oleic acid) was also widespread, reflected by the detection of C18:1 alkene (“octadecene”) in many samples. In addition, a range of mid- to long-chain alkanes (C11 to C34) were identified in almost every sample (Table 4), likely as degradation products of fatty acids or waxes. Some very long-chain compounds (C28, C31, C34, etc.) were present; these could derive from plant waxes or thermally altered lipids. Trace amounts of C20:0 fatty acid (arachidic) was indicated by C20 alkane (eicosane), and minor unsaturated chains C20:1, C22:1, etc., were occasionally noted. All samples contained a broadly similar lipid spectrum, with varying intensities but no unique compounds in any single vessel. This suggests that the pottery had been used to process a mixture of foodstuffs over its use-life, resulting in a composite residue of ubiquitous fats. Indeed, palmitic, stearic, and oleic acids are ubiquitous in both animal fats and vegetable oils. Such common fatty acids alone cannot distinguish whether a residue came from, for example, ruminant adipose fat, dairy, or plant oil. No unequivocal biomarkers were detected: notably, cholesterol (a sterol indicative of animal fat) was not identified above trace level, nor were plant-specific sterols like stigmasterol. The lack of these diagnostic compounds is likely due to both the derivatization method (which did not derivatize sterols for detection) and their possible low abundance or degradation. Likewise, we did not detect odd-chain or branched fatty acids that might hint at ruminant dairy (such as C15:0, phytanic acid) – the analysis was focused on the total lipid extract without a separate polar fraction analysis, which could have identified such markers.

**Table 4:** Major lipid residues identified by GC-MS in the pottery samples. Compounds are listed (as their hydrocarbon chain equivalents after derivatization), grouped into saturated hydrocarbons (from saturated fatty acids) and unsaturated hydrocarbons (from unsaturated fatty acids). The presence of each compound in the 54 samples is indicated by sample ID. (For brevity, this table is abbreviated; full data of all compounds vs. samples is provided in Supplementary Information).

SU	Inv. no	Sample no.	Octadecene (Oleic Acid)	Eicosane (Arachidonic Acid)	Hexadecene (Palmitic Acid)
**SU 9**	P05/110	1	X	X	
**SU 1**	USF 28	8	X	X	
**SU 1**	USF 44	14	X	X	
**SU 2**	USF 48	15	X	X	X
**SU 6**	USF 103	42	X	X	
**SU 7**	USF 104	43	X	X	
**SU 7**	USF 105	44		X	
**SU 7**	USF 109	46	X	X	
**SU 7**	USF119	52	X	X	
**US 7**	USF 121	54	X		

While the lipid evidence indicates that all vessels were used to process lipid-containing substances, it does not by itself differentiate animal vs. plant origin in this case. As shown above, the predominant lipids (C16:0, C18:0, C18:1) are common to both animal fats (adipose or dairy) and many plant oils (e.g., olive oil is high in oleic acid). The high prevalence of oleic acid (octadecene, C18:1) could suggest plant oil (like olive) usage, since oleic acid is particularly abundant in olive oil. However, oleic acid is also abundant in animal adipose tissues (e.g., pork lard, horse fat) and in some dairy fats. Likewise, palmitic and stearic acids are universal in animal fats and present in many plant fats (palm oil, etc.). However, the presence of Arachidonic acid 20:4(ω−6) (AA) could lead us to an animal origin of the fatty acids detected. Arachidonic acid is a polyunsaturated omega-6 fatty acid commonly found in animal tissues, particularly in the liver, brain, and glandular organs. Its presence in the samples listed in Table 4 suggests an origin from animal fats or phospholipids, as AA is a significant component of animal cell membranes. This finding aligns with previous studies that have identified AA as a marker for animal-derived residues in archaeological contexts [73,74]. For instance, research has demonstrated that the detection of AA in ancient pottery is indicative of the processing or storage of animal products. Therefore, the identification of arachidonic acid in these samples could support the hypothesis of animal fat utilization in the associated archaeological context. No other specific biomarker such as *β*-sitosterol (for plant oils) or diagnostic diacylglycerol patterns (for milk fats) was detected. A complete list of chromatograms is reported in S1-S40 Figs.

Crucially, however, the lipid results do not contradict an animal origin for some portion of the residues. The fatty acid distribution is entirely consistent with animal fats being present [75,76]. The lack of exclusive plant markers means we cannot exclude that the lipids came at least partly from animal products. In fact, many of the most common compounds (palmitic, stearic) tend to be relatively enriched in animal fats compared to most seed oils (olive oil being an exception with high oleic). It is reasonable to infer that animal fats were indeed present in these vessels, even though plant oils or other substances could have been contributors as well. This interpretation is strengthened when we consider the presence of AA with the proteomic data: the identification of horse albumin provides *taxonomic specificity* that lipids lack [77]. Because the proteomics conclusively demonstrates an *Equus* (likely horse) input, we can argue that at least some of the lipid residue was derived from horse meat or blood as well. Horse adipose tissue, marrow, and muscle would yield fatty acids (in particular AA) in proportions overlapping with what we observe. For instance, horse adipose fat is rich in oleic and palmitic acids (similar to other non-ruminant animals), so cooking horse meat could deposit those acids. Therefore, while the lipid analysis alone could not determine the exact contents of the vessels, the proteomic evidence for horse allows us to link the lipids to a horse meat/blood origin with much greater confidence.

Our study exemplifies how combining proteomic and lipidomic techniques provides a more powerful interpretative framework than either would alone. Lipid analysis of absorbed residues has revolutionized the study of ancient diets, but it often lacks taxonomic specificity, especially when residues result from mixed or common foodstuffs. Proteomic analysis, on the other hand, offers the possibility of species-specific and even tissue-specific identification, as we have shown by pinpointing horse serum albumin. However, proteins in archaeological ceramics can be rare and differential preservation may bias which proteins are found. By integrating both lines of evidence, we achieve a more holistic understanding that several archaeological studies have benefited from [64,73,74,78–85].

## 6. Conclusions

The present contribution provides the first direct biomolecular evidence of horse meat or blood consumption in Early Bronze Age Sicily, marking a pivotal discovery for Mediterranean archaeology. Through the combined application of proteomics and lipidomics to ceramic residues from the Marchese locality at Polizzello Mountain, we have demonstrated that vessels were used in the preparation and possible ritual offering of equine-derived products. The detection of equine serum albumin in nearly half of the analyzed samples, many with multiple unique peptides and extensive sequence coverage, represents unambiguous evidence for the processing or consumption of horse meat or blood. These findings are further reinforced by lipid profiles indicative of animal-derived fatty substances, including arachidonic acid, supporting the conclusion that horse-derived contents were present in the pottery.

These biomolecular data have a very remarkable impact on the archaeological theory of the development of the Early Bronze Age communities of Sicily. Despite the sporadic appearance of equid bones in prehistoric Sicilian assemblages and widespread doubts about their stratigraphic integrity, our results firmly situate horses within both the dietary and ritual economy of EBA Castelluccian human groups. This overturns prior assumptions that horses were either absent from the island. Instead, our evidence reveals a more nuanced scenario in which horses, possibly imported from mainland Italy, were initially incorporated into both daily consumption and elite or ceremonial practices. Notably, the occurrence of equine albumin in vessels associated with both a domestic context (the stone enclosure) and a symbolically charged ritual niche within a conical boulder suggests a continuity between household and ceremonial domains of horse use.

The ritual dimensions of these findings are particularly compelling. The carefully constructed stone enclosure, the prominently positioned boulder with a carved niche, the discovery of the terracotta phallus, the sets of table and cooking ware indicate a ceremonial space likely linked to fertility and community identity. The proteomic evidence aligns with this interpretation, suggesting the offering or consumption of horse products was not purely subsistence-driven but may have held symbolic meaning, perhaps in the context of feasting, sacrifice, or rites of passage. These practices resonate with broader Eurasian traditions of equine ritual consumption, including those seen in Indo-European contexts, and push the appearance of such behavior in the Mediterranean back by several centuries [86]. Of particular interest is the hypothesis that alongside a “blood ritual” represented by the offering (and/or likely consumption) of horse meat there could also been a “bloodless ritual” indicated by the presence of dairy product, suggested by a terracotta strainer, and its offering (and/or likely consumption), in the context of religious performance addressed to a deity destined to remain unknown.

From a methodological standpoint, this study highlights the transformative power of a multi-omics approach in archaeology. Lipid analysis alone would have revealed general food processing activities, but without taxonomic specificity. Proteomics alone, while more precise, could be limited by protein degradation or contamination concerns. When combined, however, these techniques allow us to reconstruct a detailed narrative of vessel use and dietary practice, moving from generic fatty residues to specific ingredients. This analytical synergy strengthens both the reliability and the interpretive scope of residue analysis, underscoring its value for future investigations.

The implications extend beyond local practices at Polizzello. The presence of horse-derived residues in EBA Sicily challenges established chronological and cultural models, pointing to extra-insular contacts and the elite adoption of non-local species. It also compels a reassessment of equid domestication and integration in island economies, suggesting not only that horses were present earlier than previously assumed, but that their use was embedded in complex socio-cultural frameworks.

Furthermore, the architectural continuity between the EBA ritual enclosure and Iron Age and Archaic ceremonial spaces on the Polizzello Acropolis seems to speak to a long-lived ritual tradition centered on the site, reinforcing its role as a place of enduring communal and symbolic significance. Noteworthy it is also to recall that the earliest representation of a horse, and specifically a warrior on horseback, in the Sicilian indigenous art, dated to the 7^th^ century BCE, is a two-handled amphora with incised decoration coming from the Polizzello Mountain ([87] p. 151, fig. 7). Two apparently coincidental circumstances that may inform us about a long history of cultural conservativism.

In conclusion, our study not only documents a previously unknown facet of prehistoric Sicilian subsistence and ritual life but also illustrates how scientific analyses can unlock hidden dimensions of ancient behavior. The integration of proteomic and lipidomic evidence brings new clarity to longstanding archaeological questions and positions Sicily as a key locus in the broader narrative of early Mediterranean foodways, mobility, and ritual innovation. Future studies should expand this biomolecular toolkit across other prehistoric Sicilian contexts to evaluate whether the consumption and symbolic use of horses was a localized phenomenon or part of a wider, interconnected cultural landscape.

## Supporting information

S1 Fig Chromatogram of Sample 1. (JPG)

S2 Fig Mass spectrum of the peak at RT 13.225 min in Sample 1. (JPG)

S3 Fig Mass spectrum of the peak at RT 17.159 min in Sample 1. (JPG)

S4 Fig Mass spectrum of the peak at RT 20.992 min in Sample 1. (JPG)

S5 Fig Chromatogram of Sample 8. (JPG)

S6 Fig Mass spectrum of the peak at RT 10.177 min in Sample 8. (JPG)

S7 Fig Mass spectrum of the peak at RT 13.191 min in Sample 8. (JPG)

S8 Fig Mass spectrum of the peak at RT 17.159 min in Sample 8. (JPG)

S9 Fig Chromatogram of Sample 14. (JPG)

S10 Fig Mass spectrum of the peak at RT 10.181 min in Sample 14. (JPG)

S11 Fig Mass spectrum of the peak at RT 13.191 min in Sample 14. (JPG)

S12 Fig Mass spectrum of the peak at RT 17.159 min in Sample 14. (JPG)

S13 Fig Chromatogram of Sample 15. (JPG)

S14 Fig Mass spectrum of the peak at RT 10.177 min in Sample 15. (JPG)

S15 Fig Mass spectrum of the peak at RT 13.196 min in Sample 15. (JPG)

S16 Fig Mass spectrum of the peak at RT 17.159 min in Sample 15. (JPG)

S17 Fig Chromatogram of Sample 42. (JPG)

S18 Fig Mass spectrum of the peak at RT 10.181 min in Sample 42. (JPG)

S19 Fig Mass spectrum of the peak at RT 13.191 min in Sample 42. (JPG)

S20 Fig Mass spectrum of the peak at RT 17.159 min in Sample 42. (JPG)

S21 Fig Chromatogram of Sample 43. (JPG)

S22 Fig Mass spectrum of the peak at RT 7.061 min in Sample 43. (JPG)

S23 Fig Mass spectrum of the peak at RT 13.186 min in Sample 43. (JPG)

S24 Fig Mass spectrum of the peak at RT 17.154 min in Sample 43. (JPG)

S25 Fig Chromatogram of Sample 44. (JPG)

S26 Fig Mass spectrum of the peak at RT 17.149 min in Sample 44. (JPG)

S27 Fig Mass spectrum of the peak at RT 19.003 min in Sample 44. (JPG)

S28 Fig Mass spectrum of the peak at RT 22.037 min in Sample 44. (JPG)

S29 Fig Chromatogram of Sample 46. (JPG)

S30 Fig Mass spectrum of the peak at RT 10.172 min in Sample 46. (JPG)

S31 Fig Mass spectrum of the peak at RT 13.186 min in Sample 46. (JPG)

S32 Fig Mass spectrum of the peak at RT 17.159 min in Sample 46. (JPG)

S33 Fig Chromatogram of Sample 52. (JPG)

S34 Fig Mass spectrum of the peak at RT 10.172 min in Sample 52. (JPG)

S35 Fig Mass spectrum of the peak at RT 13.191 min in Sample 52. (JPG)

S36 Fig Mass spectrum of the peak at RT 17.154 min in Sample 52. (JPG)

S37 Fig Chromatogram of Sample 54. (JPG)

S38 Fig Mass spectrum of the peak at RT 10.172 min in Sample 54. (JPG)

S39 Fig Mass spectrum of the peak at RT 13.186 min in Sample 54. (JPG)

S40 Fig Mass spectrum of the peak at RT 17.154 min in Sample 54. (JPG)
