# Supplementary figures and images for: Unearthing prehistoric diets: First evidence of horse meat consumption in Early Bronze Age Sicily
Source: PLoS One. 2025 Aug 29;20(8):e0330772. doi: 10.1371/journal.pone.0330772 (PMC12396755; doi:10.1371/journal.pone.0330772)

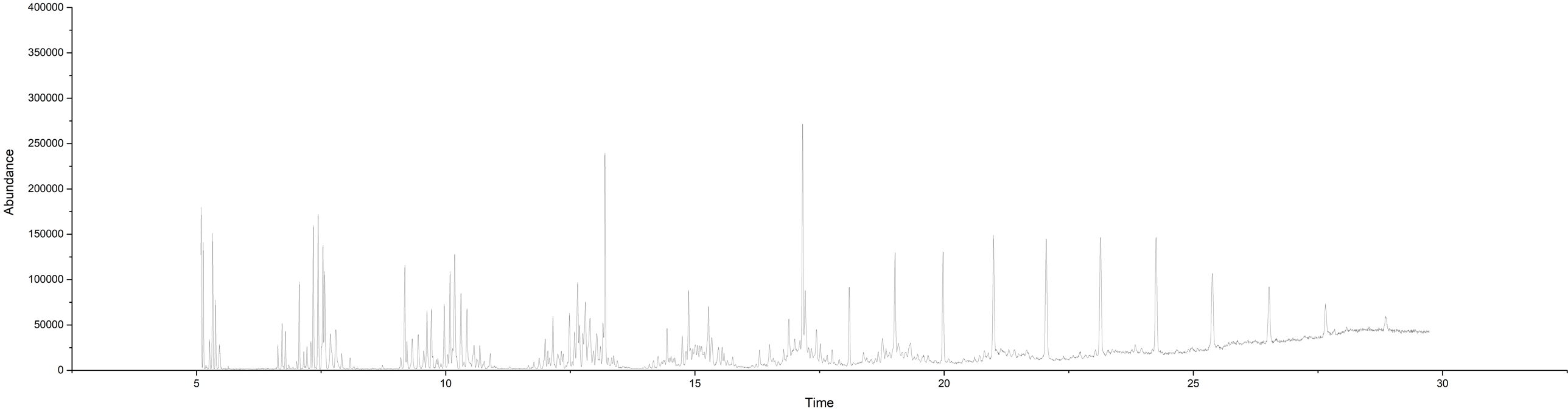

Supplement: S1 Fig — (JPG) [file pone.0330772.s001.jpg]

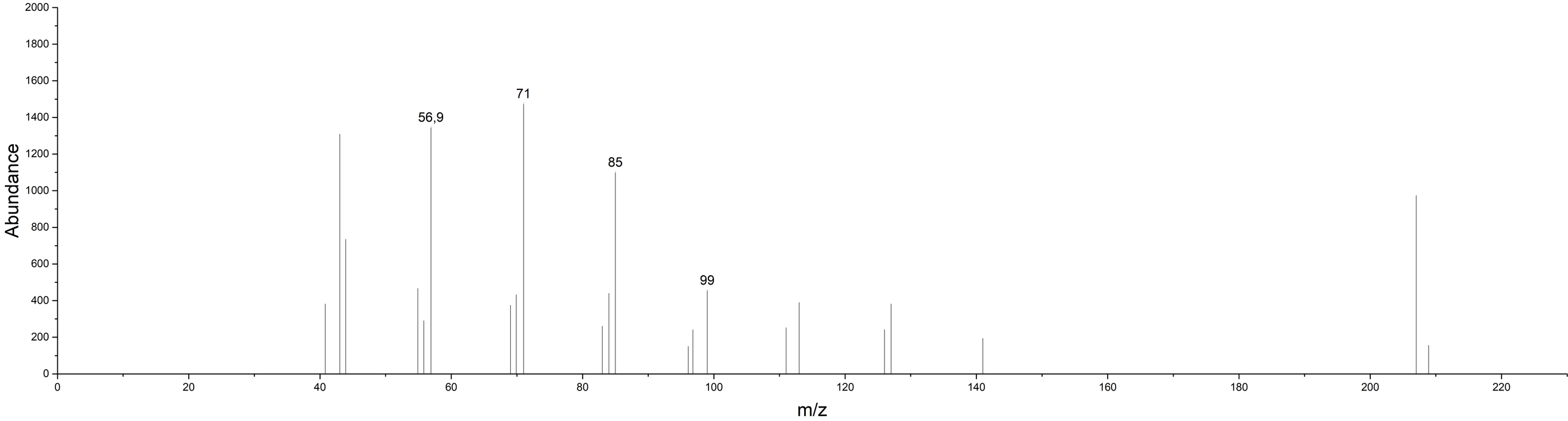

Supplement: S2 Fig — (JPG) [file pone.0330772.s002.jpg]

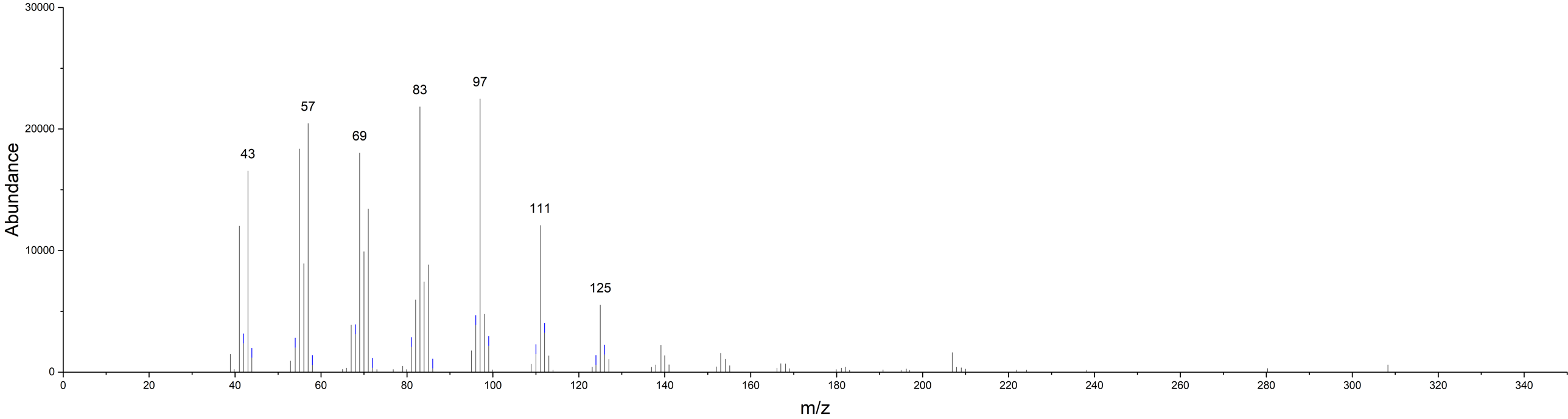

Supplement: S3 Fig — (JPG) [file pone.0330772.s003.jpg]

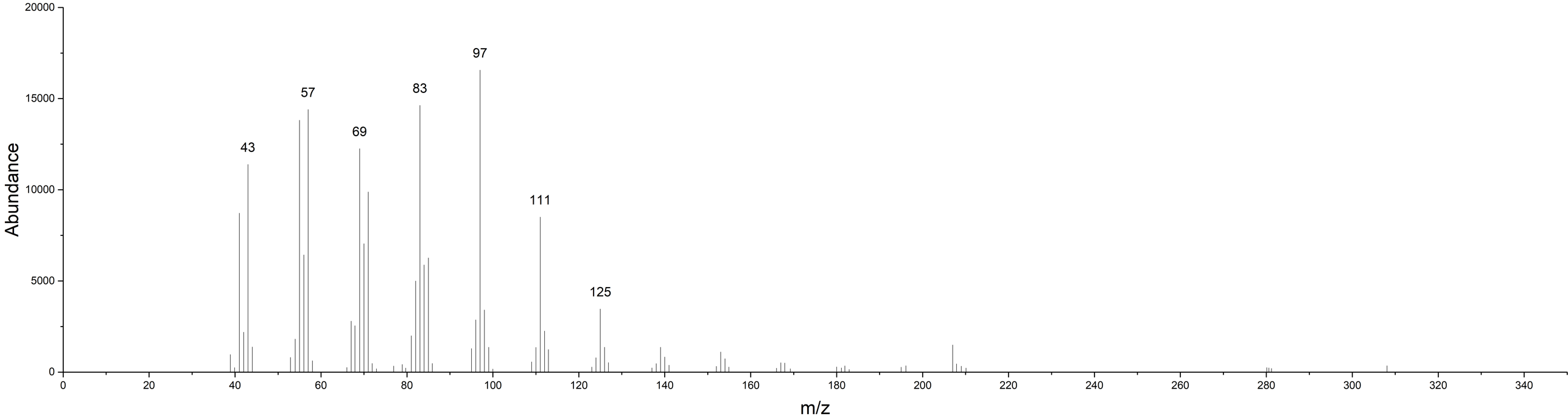

Supplement: S4 Fig — (JPG) [file pone.0330772.s004.jpg]

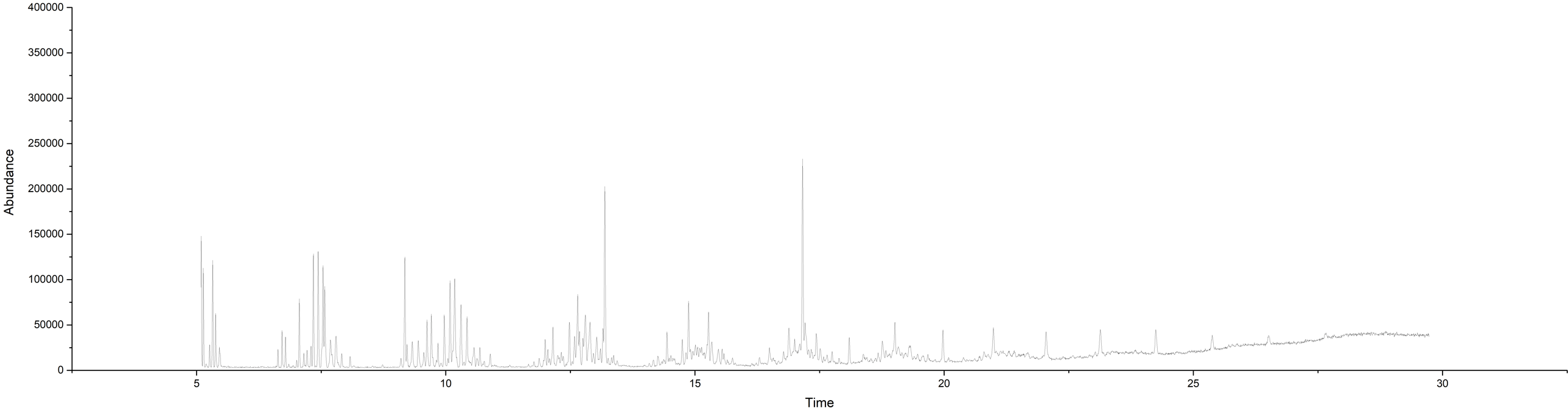

Supplement: S5 Fig — (JPG) [file pone.0330772.s005.jpg]

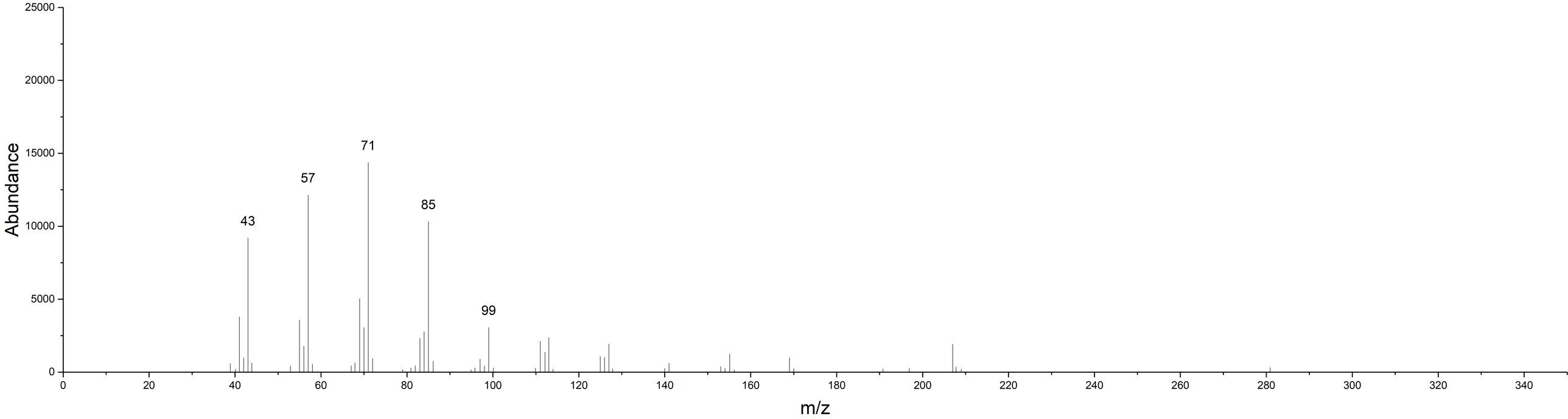

Supplement: S6 Fig — (JPG) [file pone.0330772.s006.jpg]

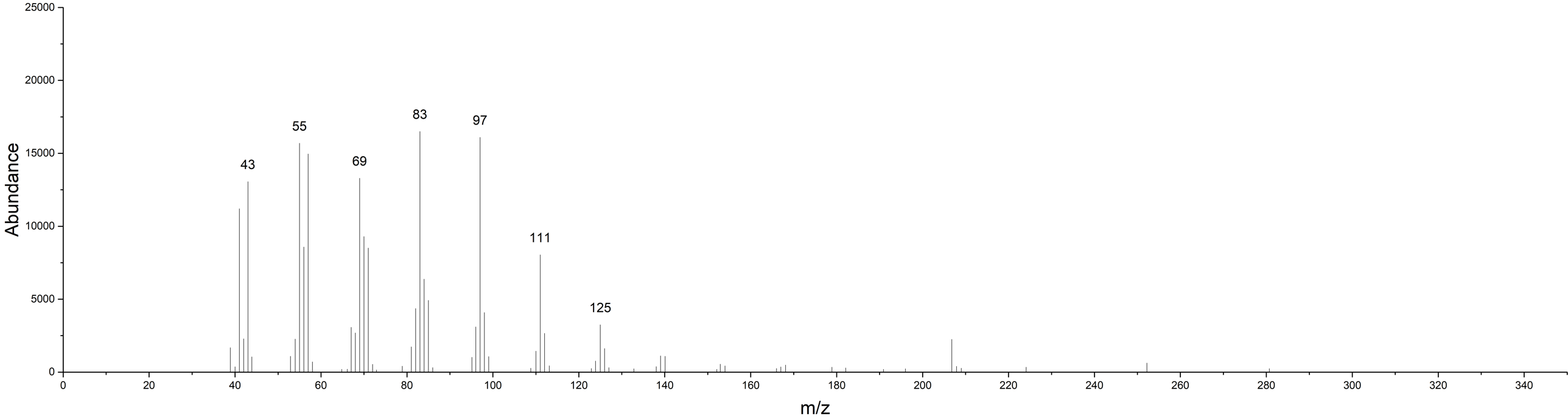

Supplement: S7 Fig — (JPG) [file pone.0330772.s007.jpg]

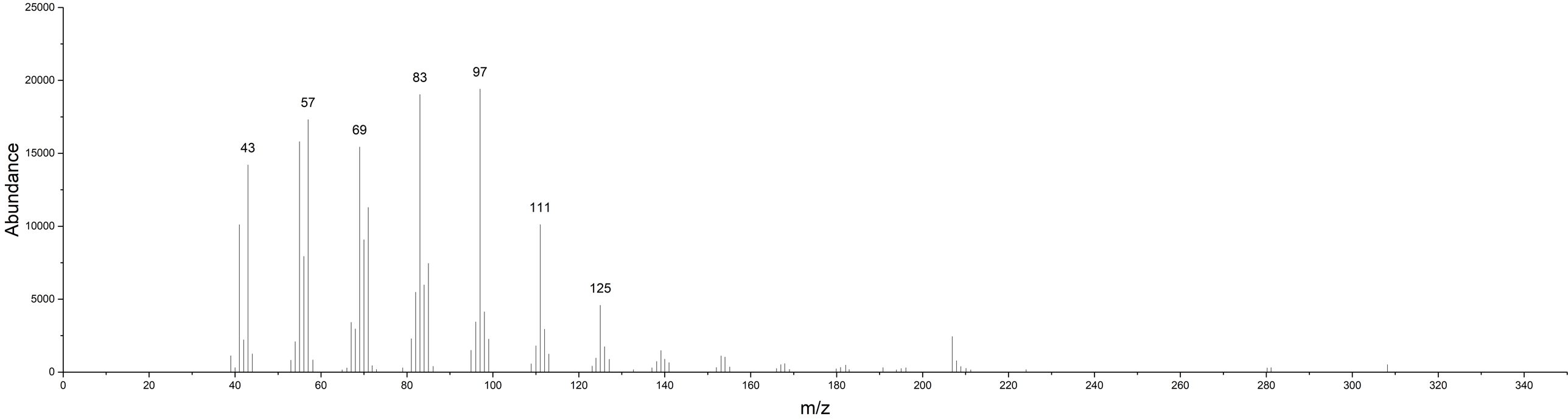

Supplement: S8 Fig — (JPG) [file pone.0330772.s008.jpg]

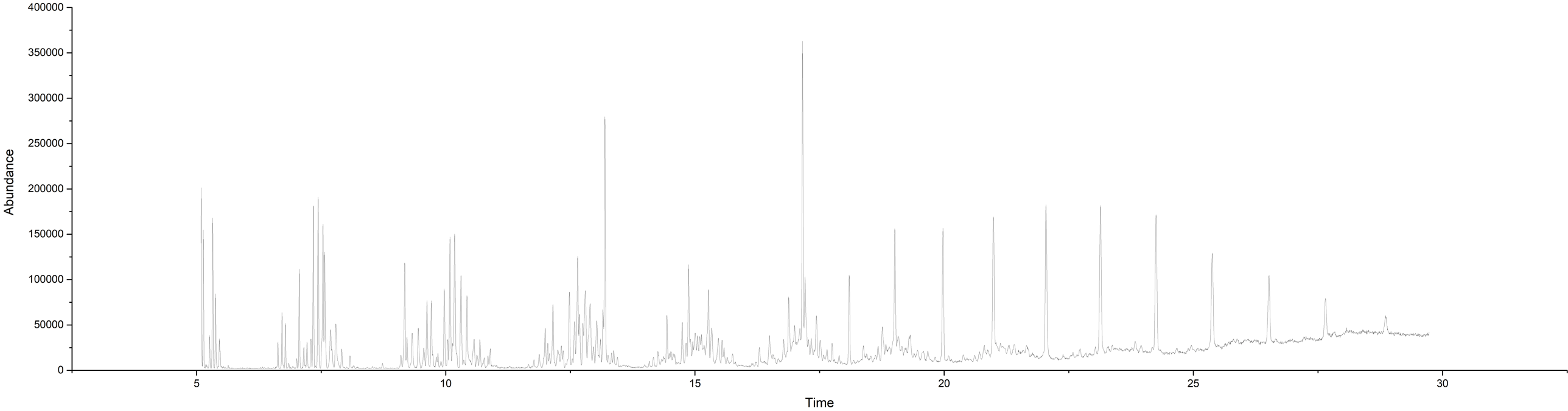

Supplement: S9 Fig — (JPG) [file pone.0330772.s009.jpg]

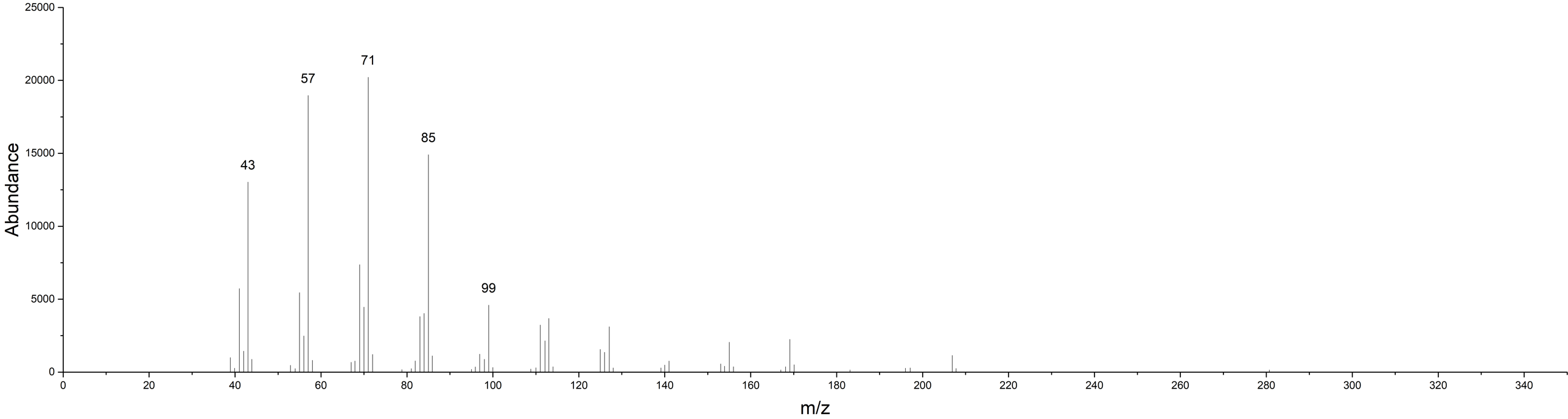

Supplement: S10 Fig — (JPG) [file pone.0330772.s010.jpg]

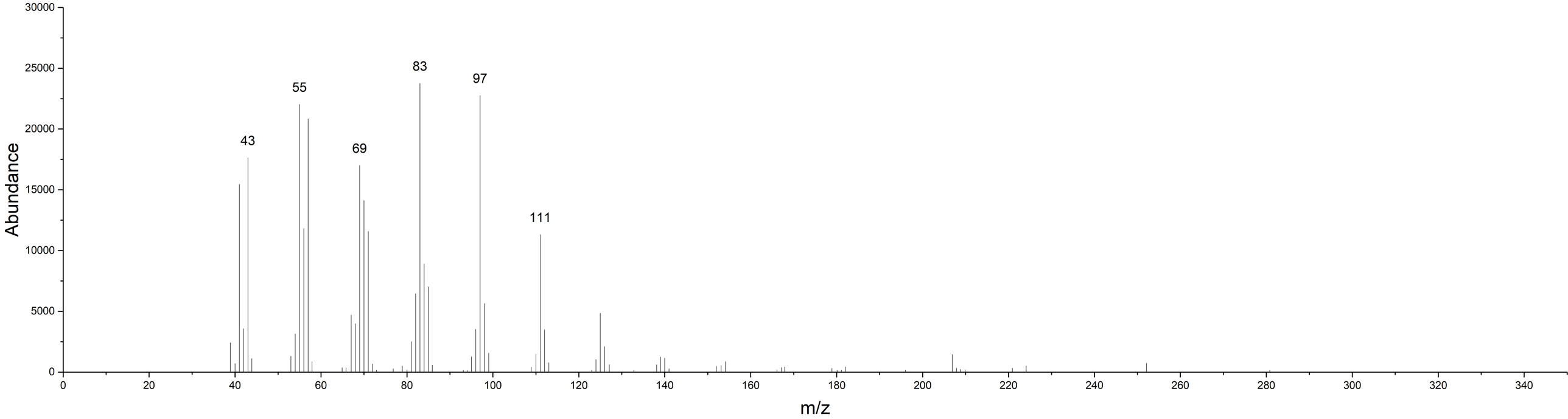

Supplement: S11 Fig — (JPG) [file pone.0330772.s011.jpg]

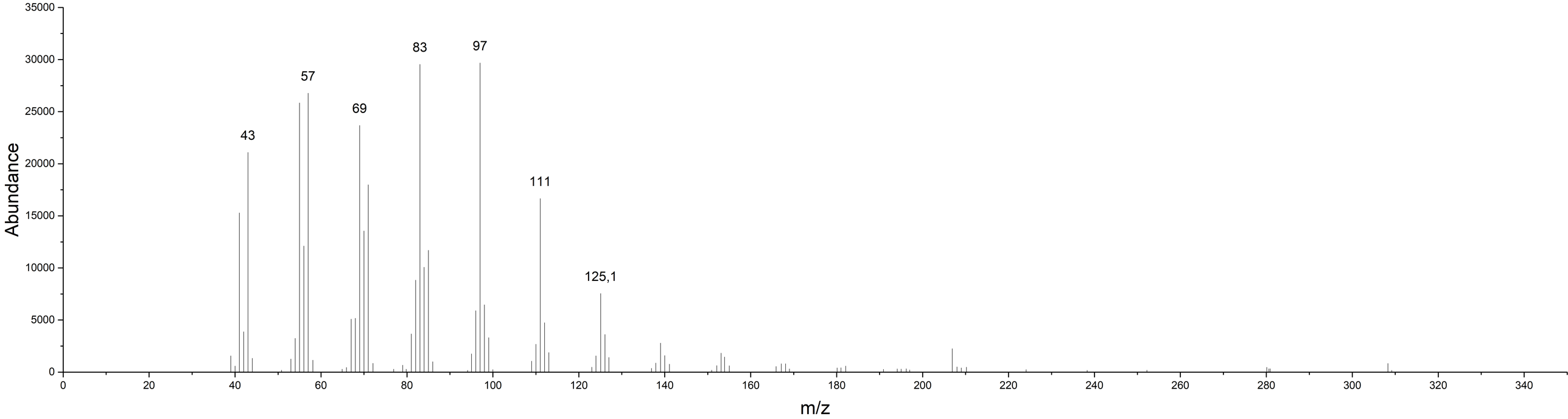

Supplement: S12 Fig — (JPG) [file pone.0330772.s012.jpg]

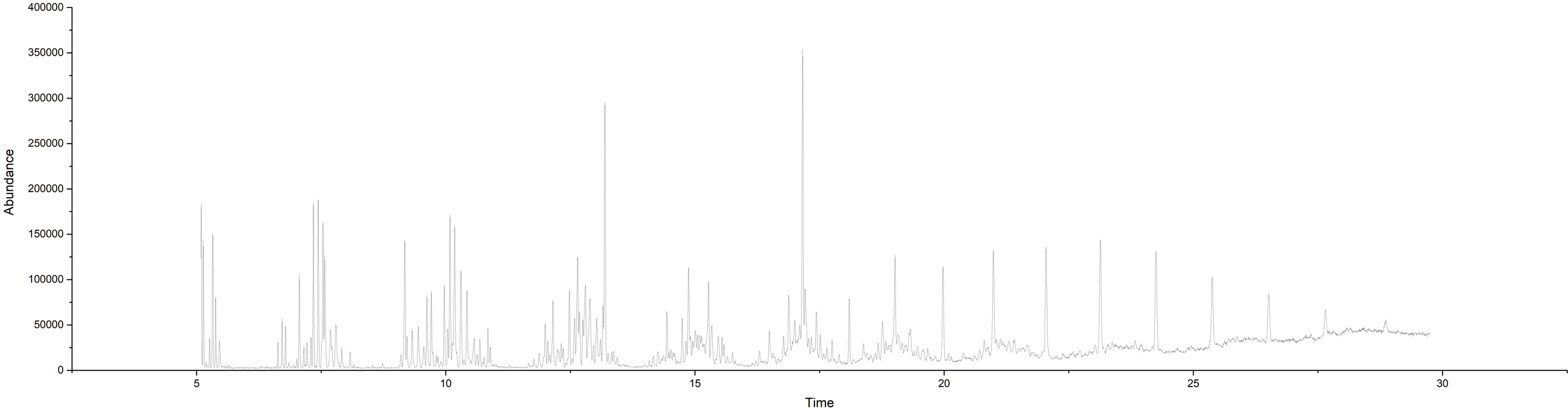

Supplement: S13 Fig — (JPG) [file pone.0330772.s013.jpg]

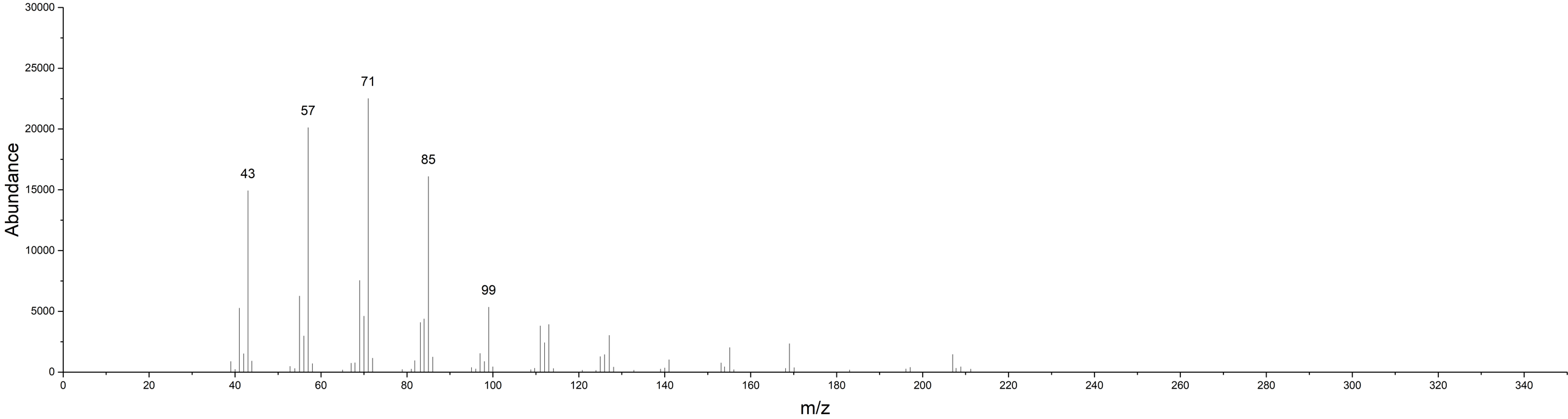

Supplement: S14 Fig — (JPG) [file pone.0330772.s014.jpg]

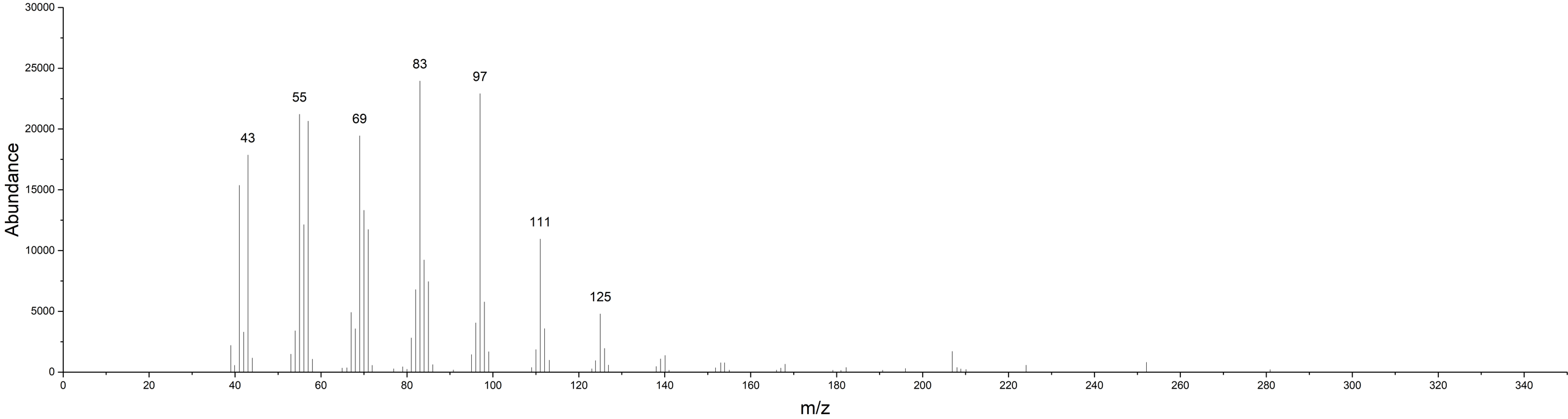

Supplement: S15 Fig — (JPG) [file pone.0330772.s015.jpg]

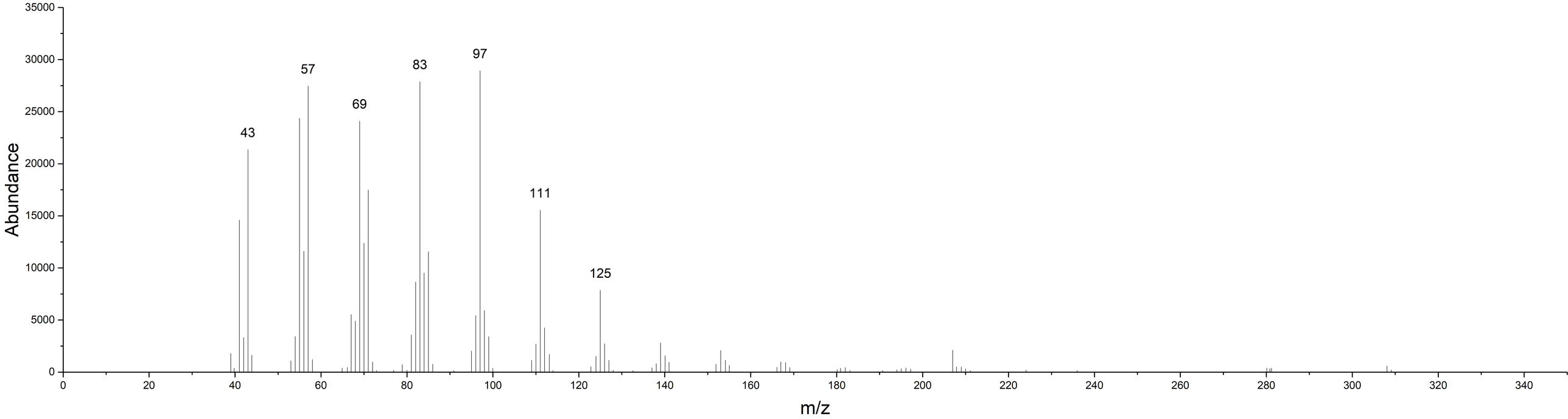

Supplement: S16 Fig — (JPG) [file pone.0330772.s016.jpg]

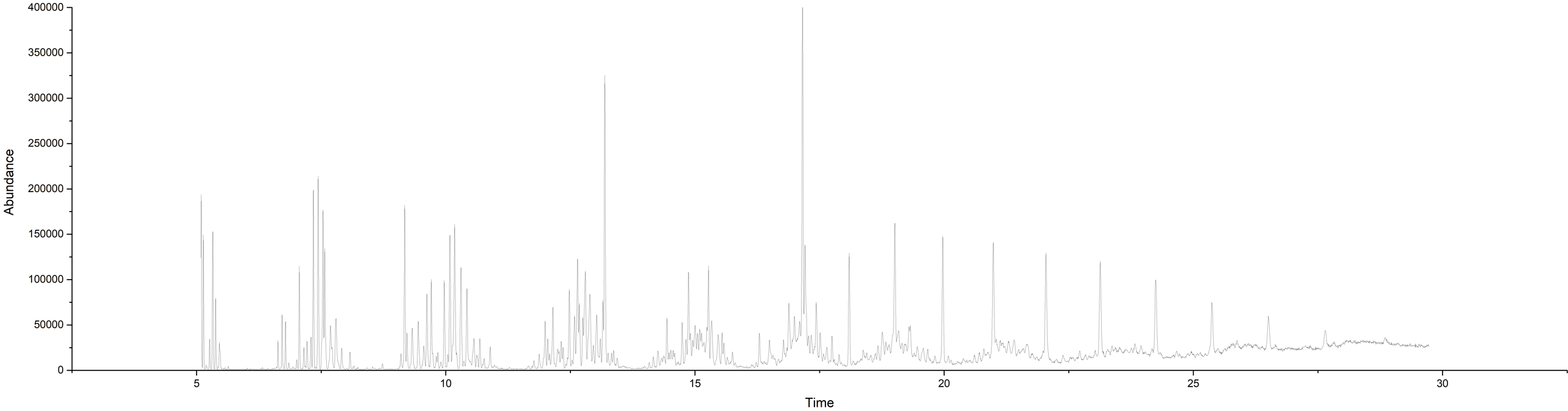

Supplement: S17 Fig — (JPG) [file pone.0330772.s017.jpg]

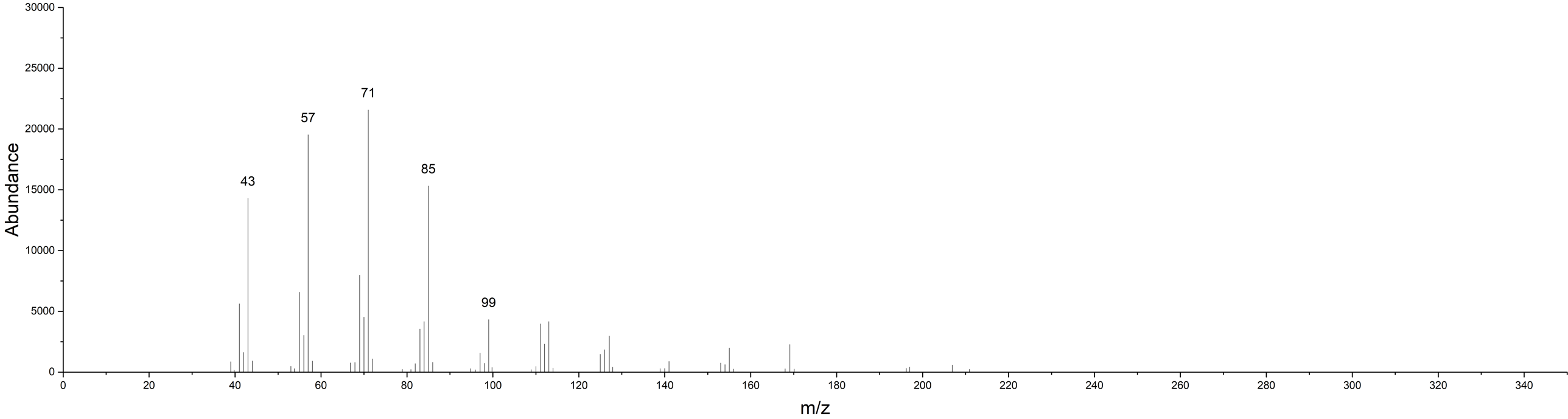

Supplement: S18 Fig — (JPG) [file pone.0330772.s018.jpg]

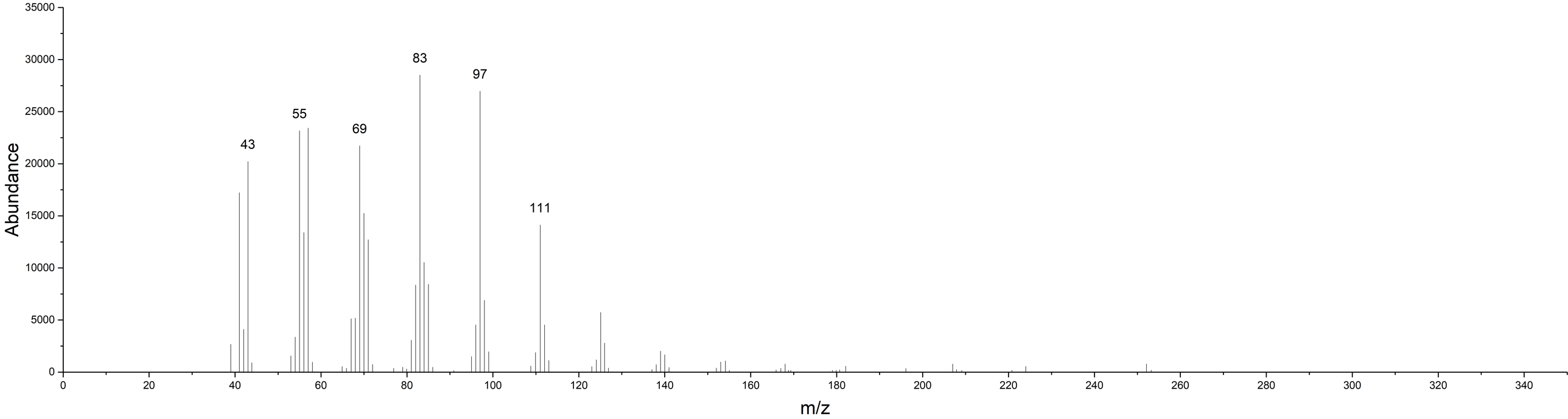

Supplement: S19 Fig — (JPG) [file pone.0330772.s019.jpg]

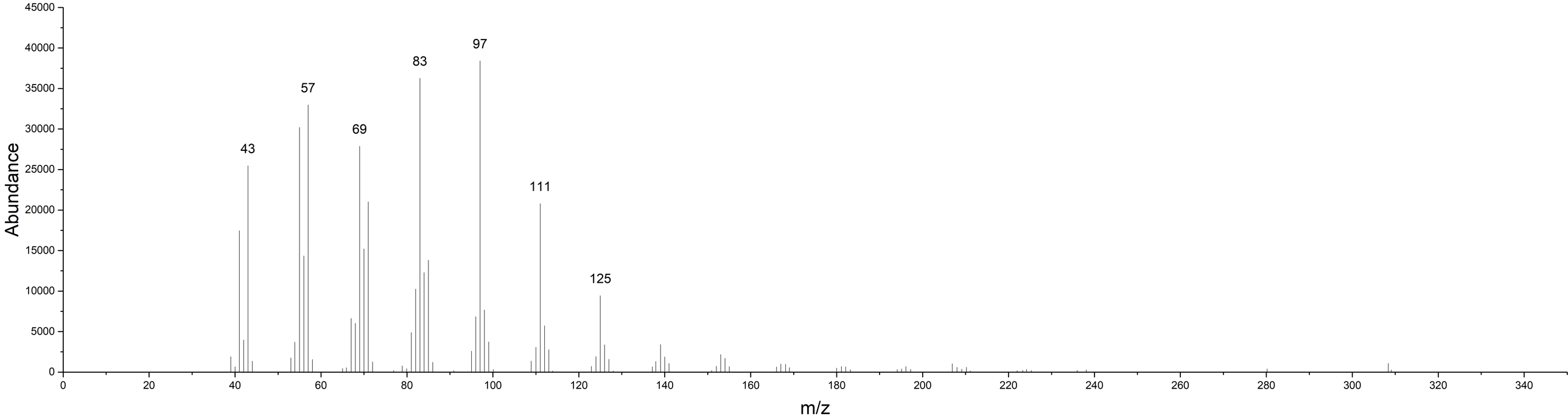

Supplement: S20 Fig — (JPG) [file pone.0330772.s020.jpg]

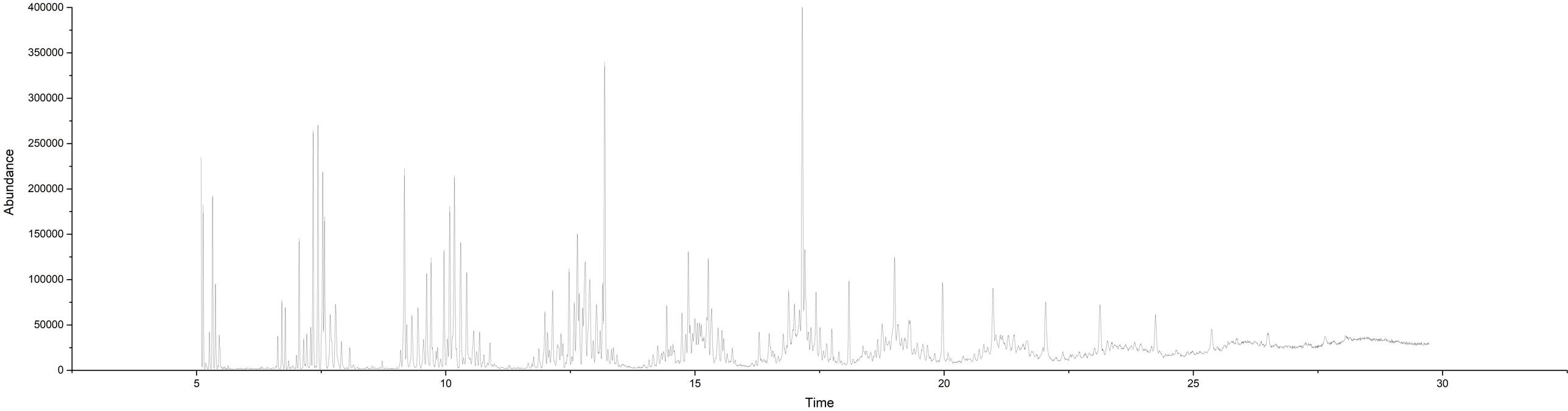

Supplement: S21 Fig — (JPG) [file pone.0330772.s021.jpg]

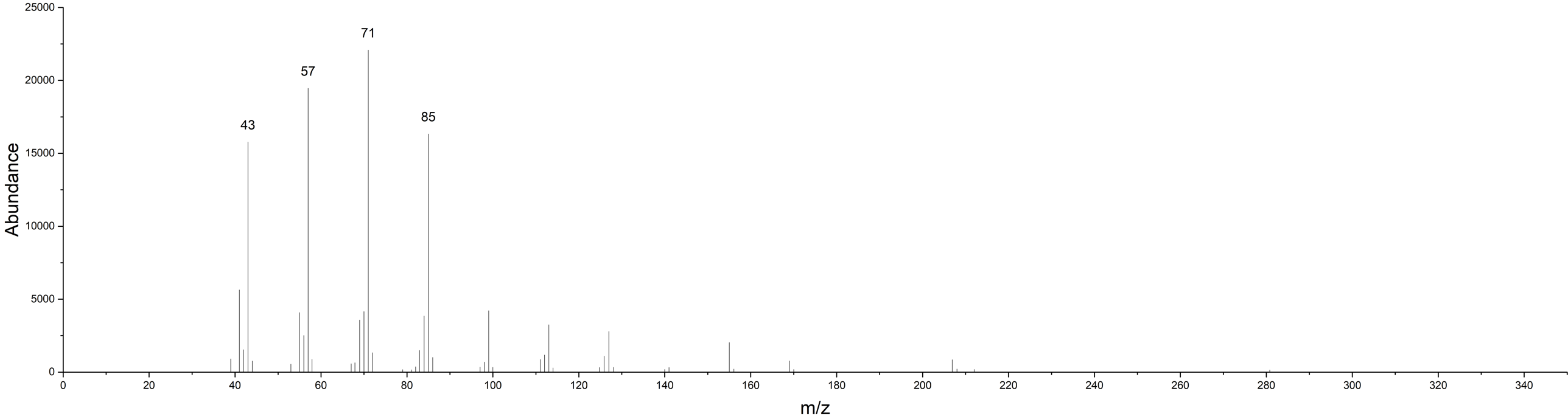

Supplement: S22 Fig — (JPG) [file pone.0330772.s022.jpg]

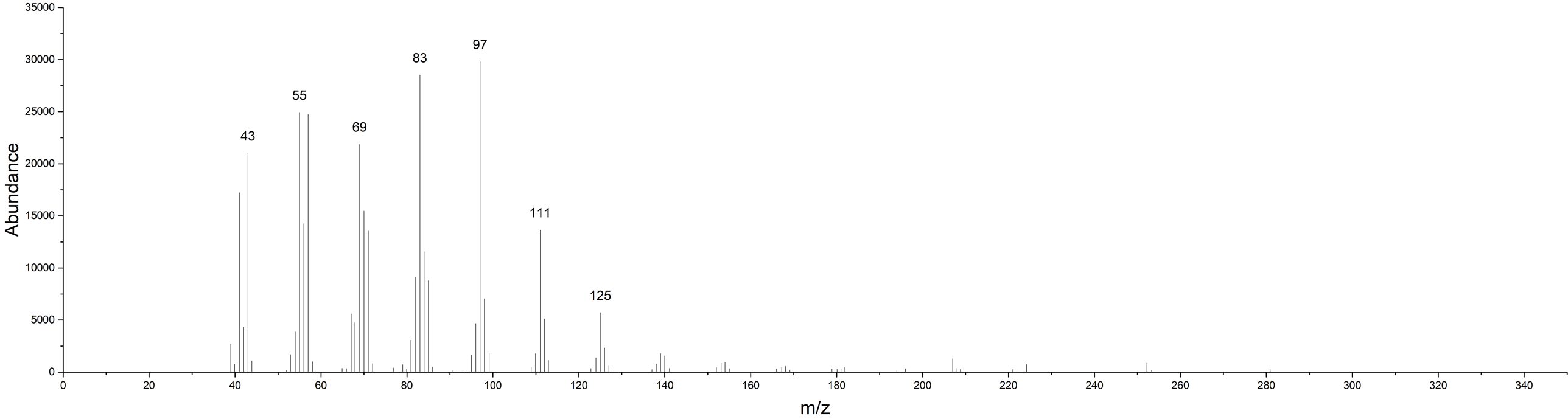

Supplement: S23 Fig — (JPG) [file pone.0330772.s023.jpg]

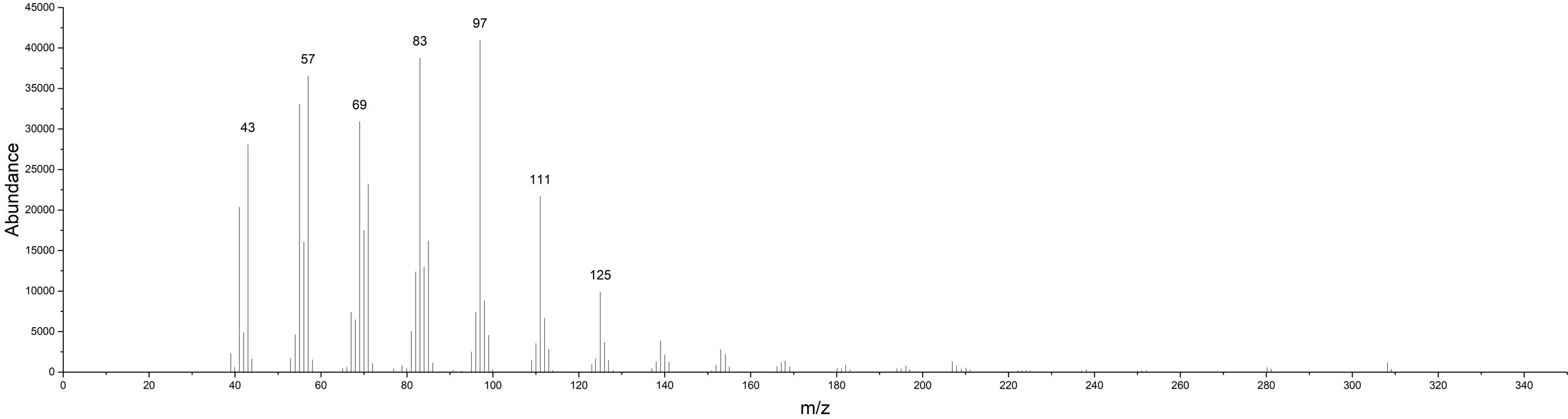

Supplement: S24 Fig — (JPG) [file pone.0330772.s024.jpg]

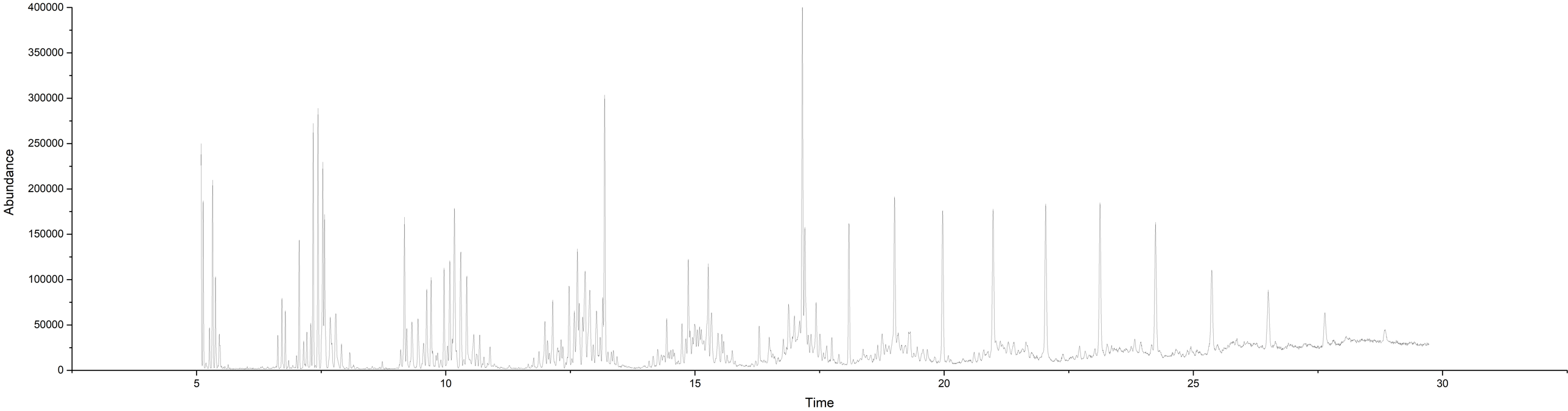

Supplement: S25 Fig — (JPG) [file pone.0330772.s025.jpg]

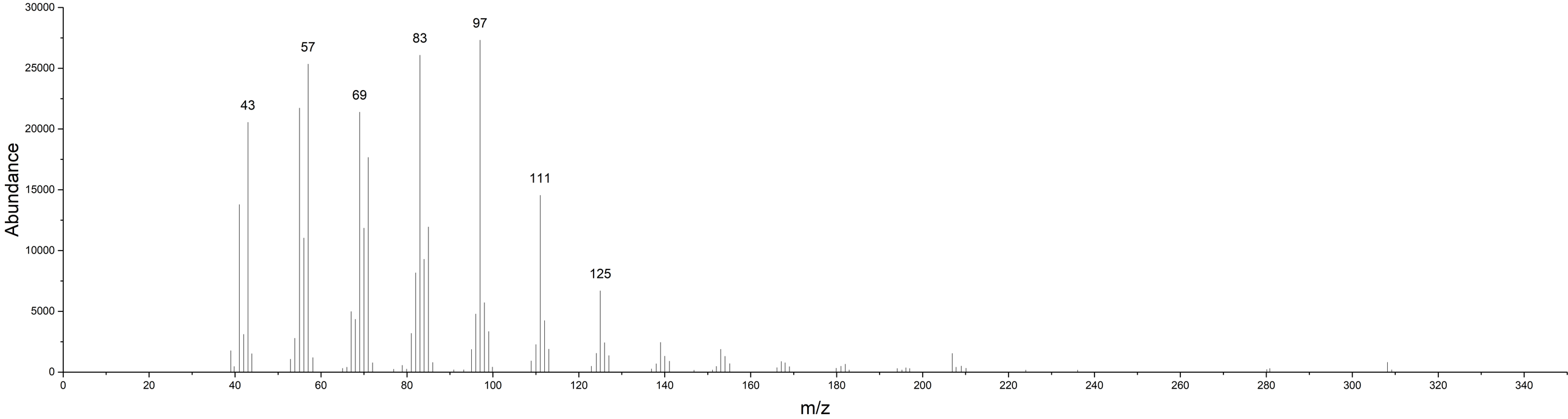

Supplement: S26 Fig — (JPG) [file pone.0330772.s026.jpg]

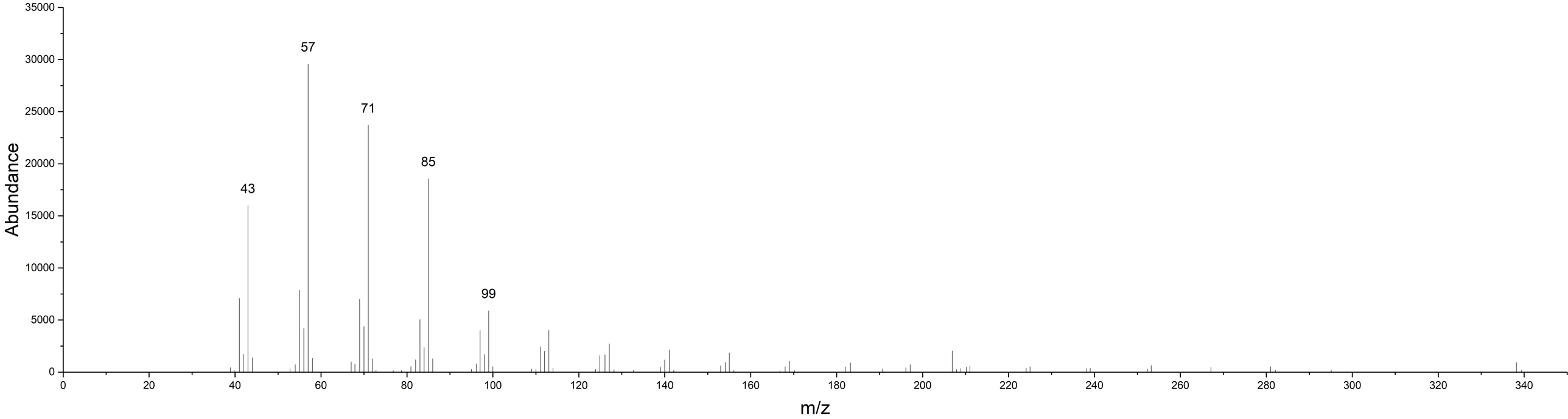

Supplement: S27 Fig — (JPG) [file pone.0330772.s027.jpg]

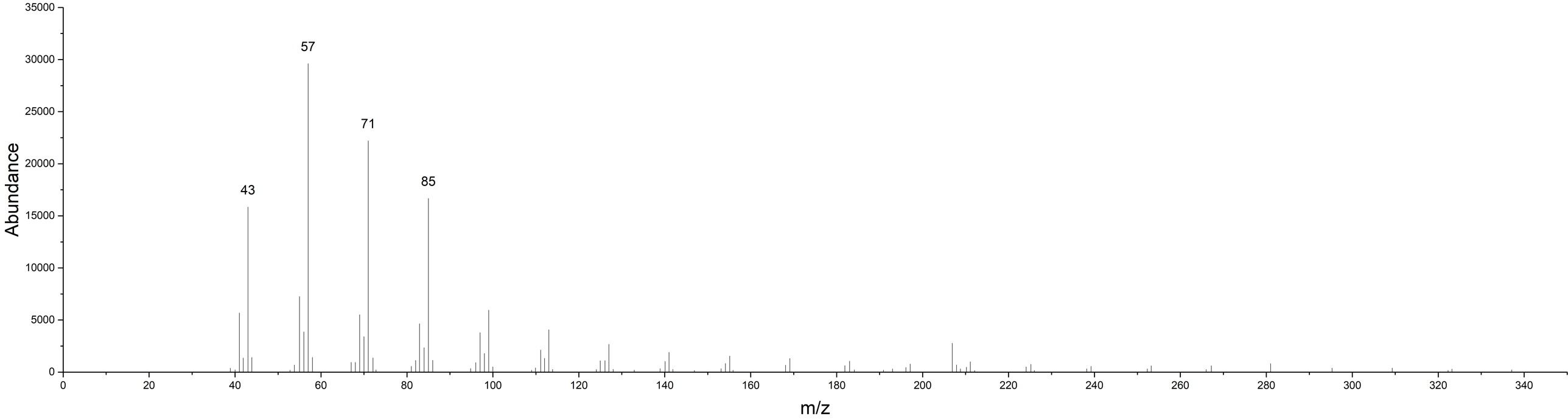

Supplement: S28 Fig — (JPG) [file pone.0330772.s028.jpg]

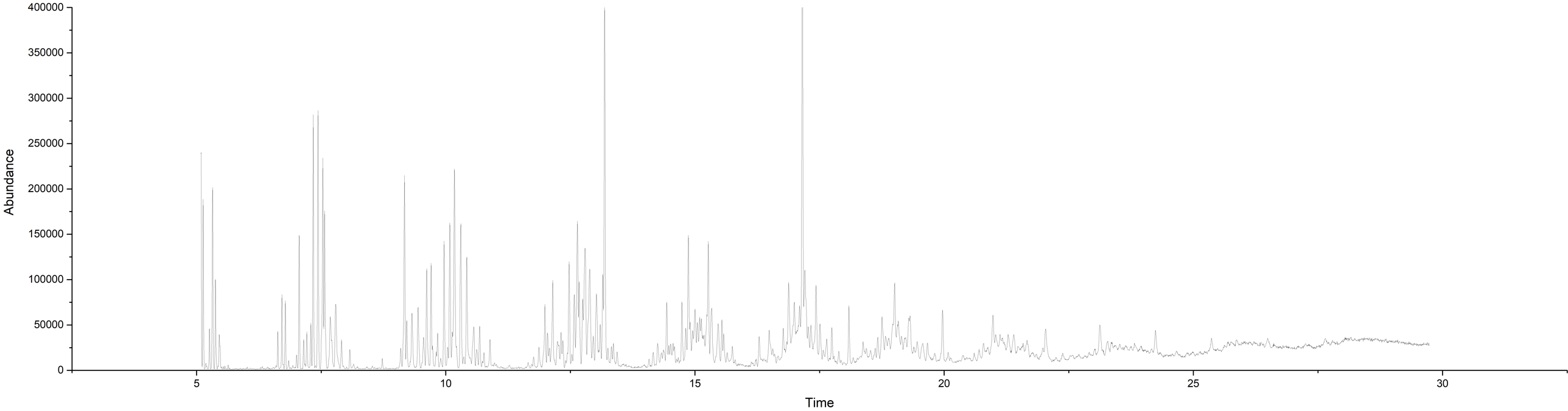

Supplement: S29 Fig — (JPG) [file pone.0330772.s029.jpg]

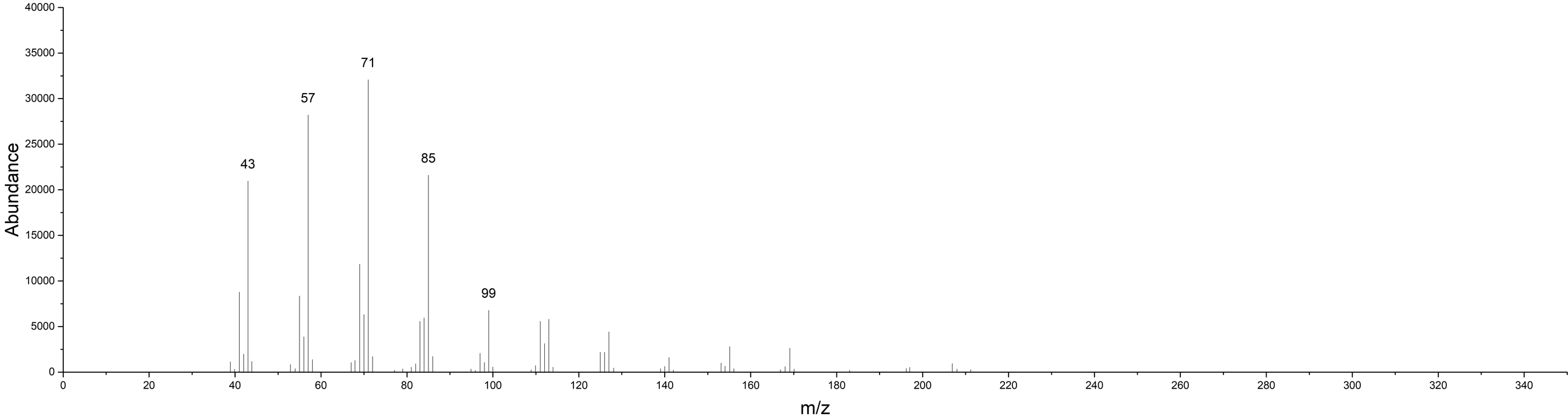

Supplement: S30 Fig — (JPG) [file pone.0330772.s030.jpg]

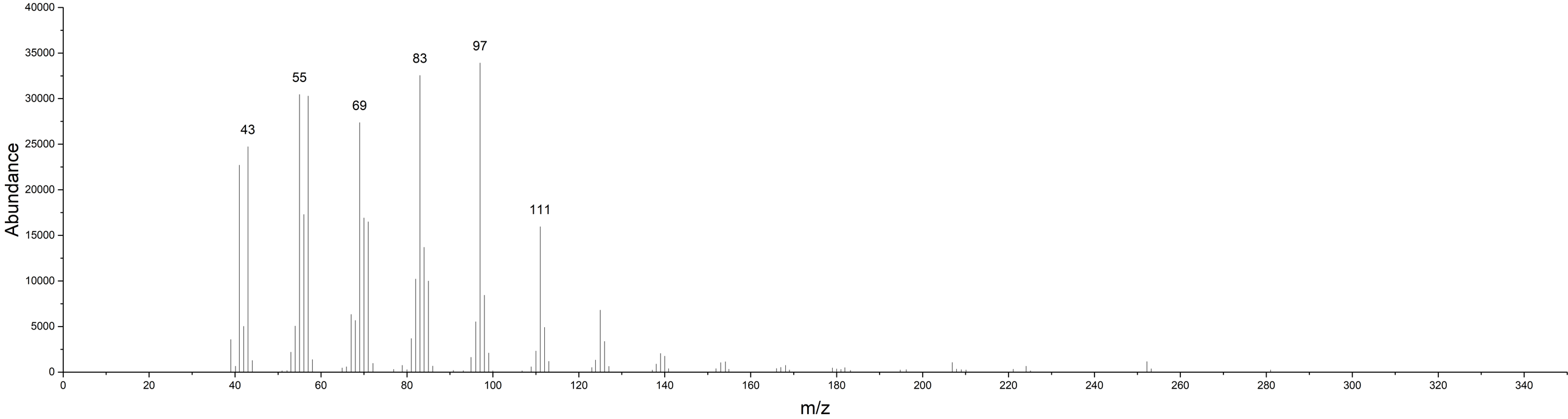

Supplement: S31 Fig — (JPG) [file pone.0330772.s031.jpg]

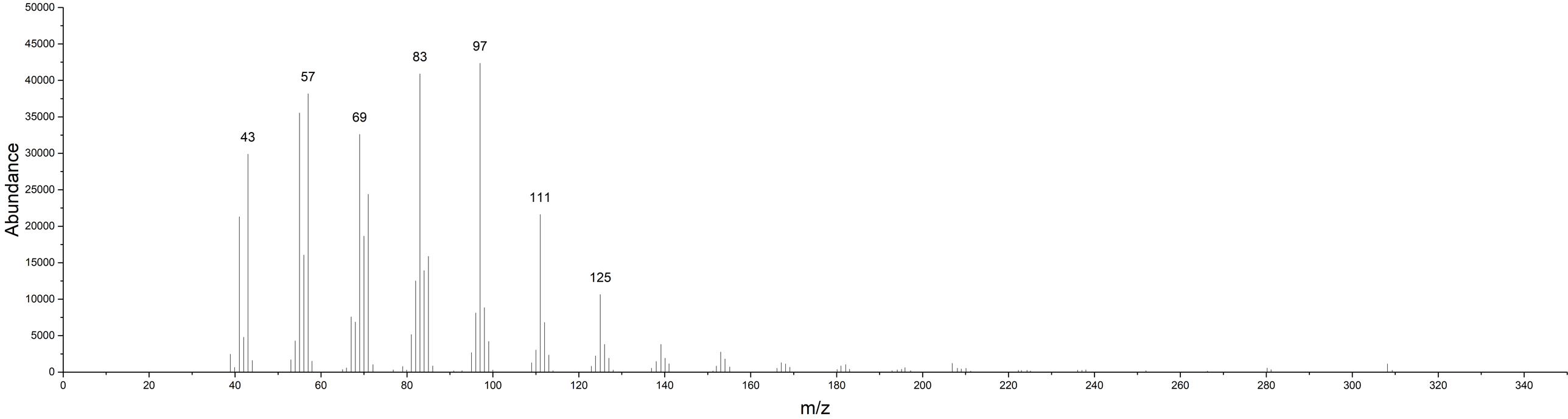

Supplement: S32 Fig — (JPG) [file pone.0330772.s032.jpg]

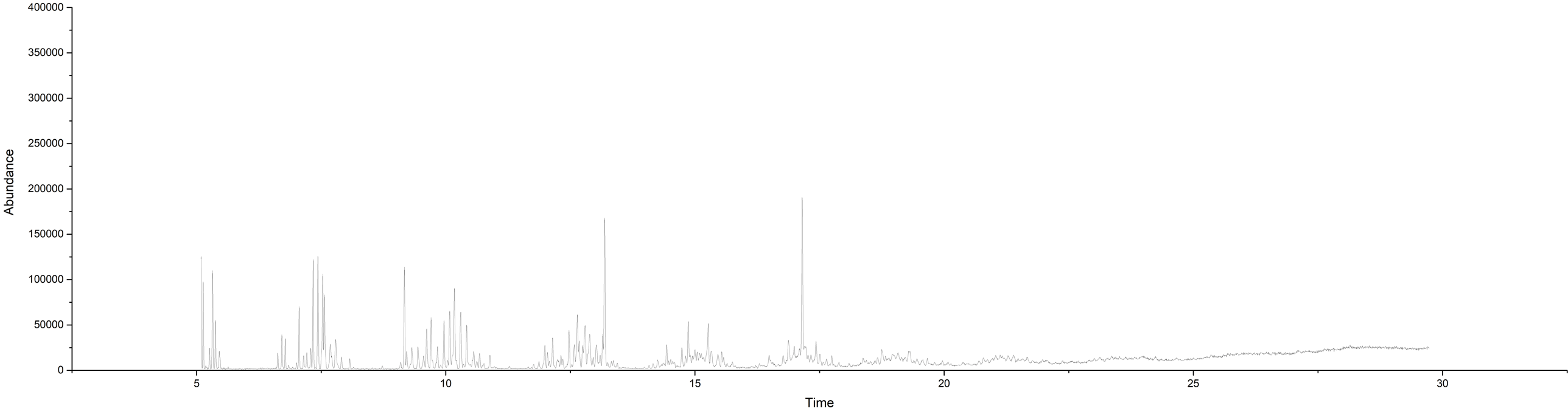

Supplement: S33 Fig — (JPG) [file pone.0330772.s033.jpg]

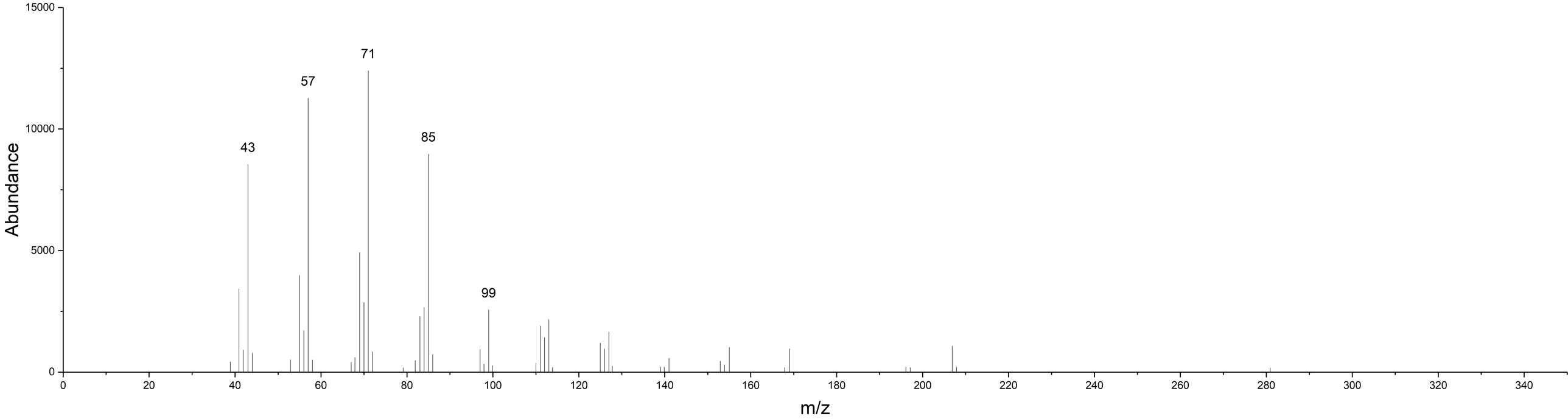

Supplement: S34 Fig — (JPG) [file pone.0330772.s034.jpg]

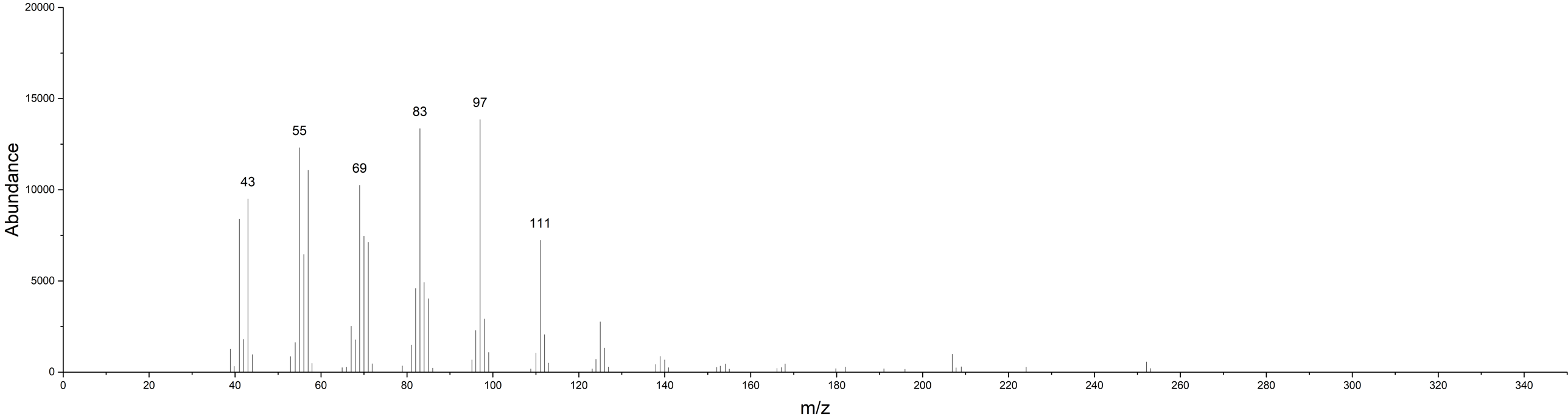

Supplement: S35 Fig — (JPG) [file pone.0330772.s035.jpg]

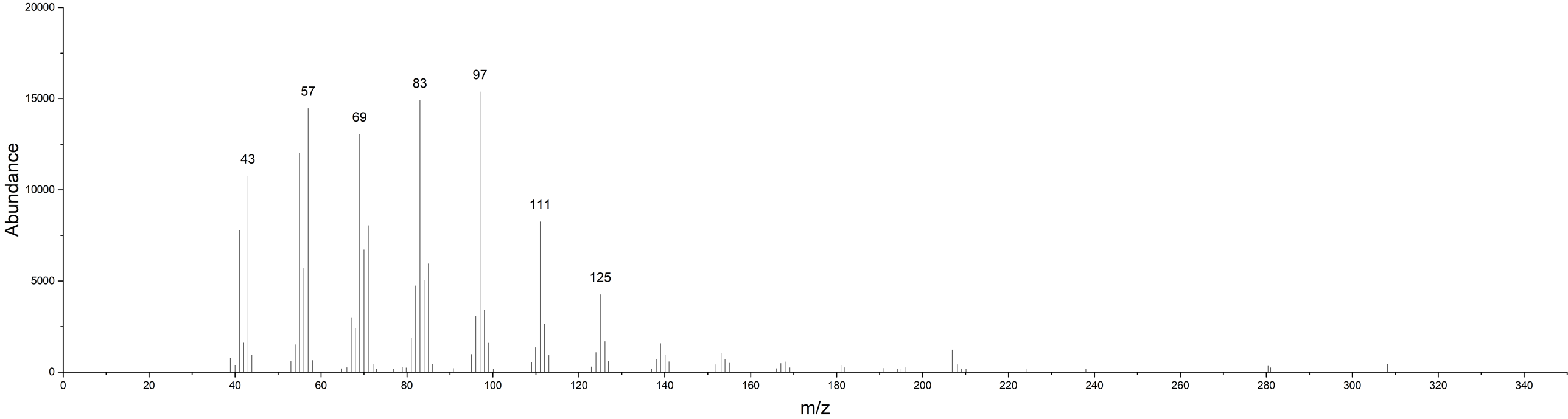

Supplement: S36 Fig — (JPG) [file pone.0330772.s036.jpg]

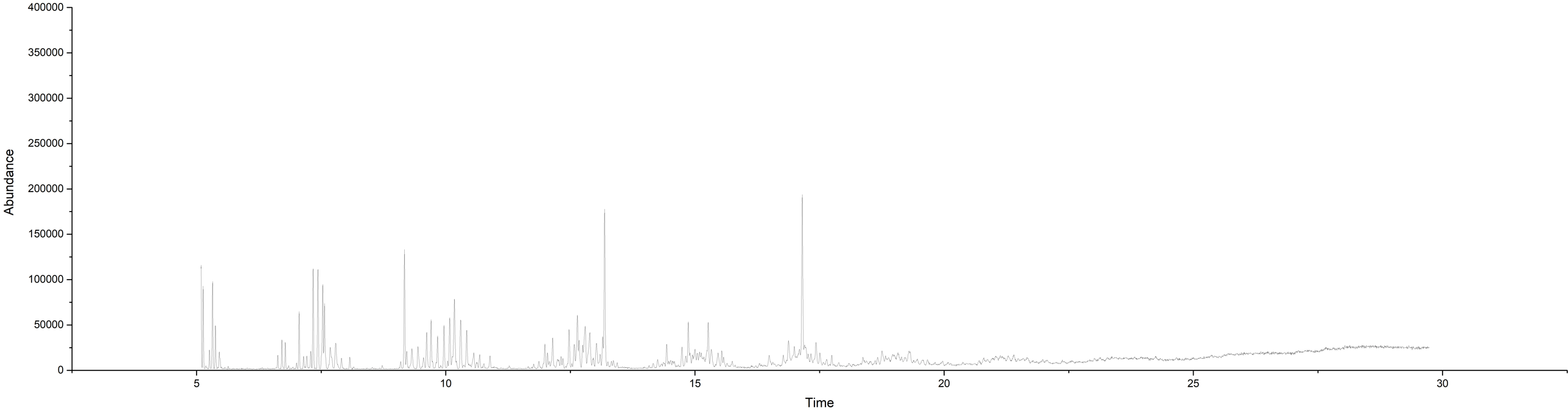

Supplement: S37 Fig — (JPG) [file pone.0330772.s037.jpg]

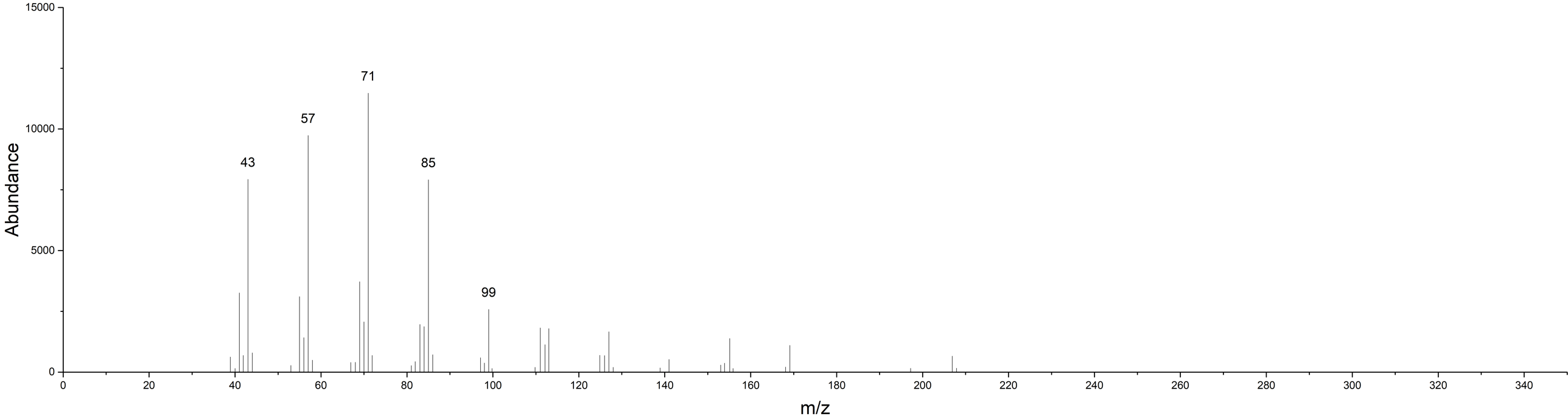

Supplement: S38 Fig — (JPG) [file pone.0330772.s038.jpg]

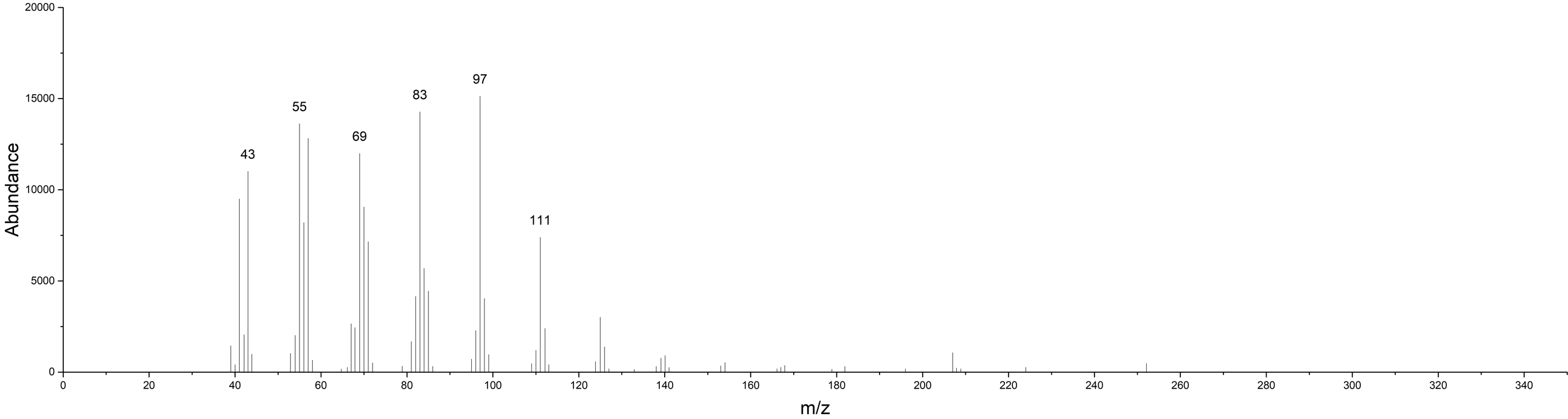

Supplement: S39 Fig — (JPG) [file pone.0330772.s039.jpg]
